# Efficacy of intermittent versus daily vitamin D supplementation on improving circulating 25(OH)D concentration: a Bayesian network meta-analysis of randomized controlled trials

**DOI:** 10.3389/fnut.2023.1168115

**Published:** 2023-08-24

**Authors:** Yan Zhuang, Zhe Zhu, Peihan Chi, Haibo Zhou, Zhicheng Peng, Haoyue Cheng, Xing Xin, Wenliang Luo, Shuting Si, Minjia Mo, Danqing Chen, Hui Liu, Yunxian Yu

**Affiliations:** ^1^Department of Public Health, Second Affiliated Hospital of Zhejiang University School of Medicine, Hangzhou, China; ^2^Department of Anesthesiology, Second Affiliated Hospital of Zhejiang University School of Medicine, Hangzhou, China; ^3^The Second School of Clinical Medicine, Southern Medical University, Guangzhou, China; ^4^Department of Obstetrics and Gynecology, Woman's Hospital, School of Medicine, Zhejiang University, Hangzhou, China; ^5^Central Lab, Sir Run Run Shaw Hospital, School of Medicine, Zhejiang University, Hangzhou, China

**Keywords:** vitamin D, daily supplementation, intermittent supplementation, 25(OH)D concentration, network meta-analysis

## Abstract

**Background:**

Vitamin D deficiency is a widespread issue globally, resulting in increased use of vitamin D supplements. However, it is unclear whether intermittent (weekly or monthly) vitamin D supplementation is as effective as daily supplementation in improving circulating 25-hydroxyvitamin D [25(OH)D] levels.

**Methods:**

Three databases including Medline, EMBASE, and the Cochrane Library were systematically searched up to 10 November 2020. The risk of bias was evaluated according to Cochrane Collaboration’s tool for rating methodological quality assessment. Direct and indirect comparisons between interventions and controls were performed by a Bayesian network meta-analysis (NMA), where the mean difference (MD) and its 95% confidence interval (CI) were used to indicate the efficacy.

**Results:**

This NMA analysis included 116 RCTs with a total of 11,376 participants. Generally, we observed that 25(OH)D concentrations were significantly elevated regardless of vitamin D supplementation frequency. Although the findings of SUCRA indicated that daily vitamin D supplementation had a higher rank value than intermittent supplementation when the supplement dosage was similar, no statistically significant pooled mean differences of 25(OH)D concentration were noted between the daily supplementation group and intermittent supplementation group. Additionally, weekly supplementation with a total of 600,000 IU vitamin D supplementation during 3 months had the best efficacy in elevating 25(OH)D concentration (pooled MD = 63 nmol/L, 95%CI: 49–77). To achieve optimal 25(OH)D concentration (>75 nmol/L), we recommend 60,000 IU vitamin D supplementation monthly (~2,000 IU/day).

**Conclusion:**

The efficacy of intermittent vitamin D supplementation was similar to daily supplementation. Coupled with its convenience, the frequency and dosage of intermittent vitamin D supplements were recommended to reach the optimal 25(OH)D level.

**Systematic review registration:**https://www.crd.york.ac.uk/prospero/display_record.php?RecordID=257257, PROSPERO CRD42021257257.

## Introduction

Vitamin D deficiency is a prevalent issue affecting various populations worldwide, with a prevalence rate ranging from 24% in the United States to 90% in the Middle East (1). Notably, vitamin D deficiency has been reported to be associated with several health problems, including skeletal diseases, cardiovascular diseases, diabetes, depression, neurodegenerative diseases, and cancer (2). The circulating 25-hydroxyvitamin D [25(OH)D] concentration is widely regarded as a reliable indicator of vitamin D status (3). Vitamin D deficiency is defined as a 25(OH)D concentration of less than 50 nmol/L, while insufficiency is defined as a 25(OH)D concentration ranging from 50 to 75 nmol/L, according to the guidelines of the United States Endocrine Society (4). Several factors contribute to vitamin D deficiency, including aging, inadequate outdoor activity, excessive sun protection, insufficient vitamin D supplementation, obesity, high latitude, and simple diet (5).

Vitamin D can be synthesized in the skin through exposure to ultraviolet B rays, as well as obtained from dietary intake or supplements. Vitamin D supplementation is a quick and easy way to improve 25(OH)D levels. The approach to vitamin D supplementation varies across randomized controlled trials (RCTs), with some studies focusing on daily supplementation (6–8), others on intermittent (weekly or monthly) supplementation (9–11), and still others on high-dose supplementation (12, 13). However, hypercalcemia, particularly with high-dose supplementation, is a major concern (14). In practice, compliance with daily supplementation can be challenging (15), making intermittent supplementation an attractive alternative if it proves to be as effective as daily supplementation while ensuring safety. To date, there is no integrated evidence on the relative effects of all forms of vitamin D supplementation.

This study aims to assess the effect of intermittent (weekly and monthly) vitamin D supplementation compared to daily supplementation on improving circulating 25(OH)D levels, under the condition of similar dosage and supplementation duration. Additionally, we aim to recommend vitamin D supplementation frequency and dosage to achieve optimal 25(OH)D concentration. The network meta-analysis (NMA) approach will be used to incorporate direct or indirect evidence (16), enabling comprehensive comparisons for all RCTs, including comparisons with placebo/control, different dosages of vitamin D supplementation, and different frequencies of vitamin D supplementation, while fully considering randomization.

## Materials and methods

### Data sources

This meta-analysis adhered to the Preferred Reporting Items for Systematic Reviews and Meta-analyses (PRISMA) extension statement for systematic reviews incorporating NMA (Supplementary Table S1) (16). We searched the MEDLINE, EMBASE, and the Cochrane Library databases from inception to 10 November 2020, without language restriction, using combinations of MESH terms or keywords related to RCTs and vitamin D supplementation. The full search strategy is presented in Supplementary Table S2. We also reviewed the reference lists of included articles and relevant systematic reviews.

### Study selection

We included RCTs that assessed the effect of vitamin D_3_ supplementation on circulating 25(OH)D concentrations, compared with controls (including placebo and blank control) or with different dosages of vitamin D_3_ in adults. We combined placebo and blank control into one group because only 9 out of 116 studies used blank control. We excluded studies involving children, pregnant women, and patients with severe liver and kidney-related diseases that affect vitamin D synthesis and metabolism. However, we included participants with diseases such as diabetes mellitus, obesity, and polycystic ovary syndrome. We also excluded studies that changed the dosage of vitamin D supplementation during the study period or used vitamin D analogs. Pairs of reviewers (Y Zhuang and Z Zhu, PH Chi and HB Zhou, ZC Peng and HY Cheng, and X Xin and WL Luo) independently performed the study selection, including screening titles and abstracts, followed by full-text evaluation. Any disagreements between reviewer pairs were resolved by a third reviewer.

### Data extraction

For each eligible study, pairs of reviewers independently extracted the following items and recorded them in a formatted Excel file: study characteristics (the first author, publication year, country/city, and latitude), population characteristics (sample size, mean age, proportion of males, disease status, and vitamin D deficiency), intervention and comparator descriptions (dose, frequency, duration, and co-supplementation with calcium), and outcomes (baseline and follow-up 25(OH)D levels or change of 25(OH)D levels). When relevant information was unclear or missing, we contacted the authors for additional information. Any disagreements within reviewer pairs were resolved by consulting a third reviewer.

### Risk of bias assessment

We independently evaluated the risk of bias (RoB) for each eligible trial using the Cochrane Collaboration’s RoB2 tool (17). The RoB2 tool includes seven domains: random sequence generation, allocation concealment, blinding of participants and personnel, blinding of outcome assessment, incomplete outcome data, selective reporting, and other sources of bias. Any disagreements between the reviewer pairs were resolved by consulting a third reviewer.

### Data synthesis

When the mean differences (MD) and standard deviations (SD) of circulating 25(OH)D levels were given, we extracted the data directly. When baseline and follow-up values were available, we estimated the MDs and SDs using the formula propounded in the Cochrane Handbook for Systematic Reviews (18). We also calculated SDs from 95%CIs. Furthermore, according to the formula described by Shi et al. (19), we converted median and ranges/interquartile ranges into means and SDs. We used nmol/L as the uniform unit of circulating 25(OH)D concentrations. The unit of circulating 25(OH)D concentration reported in ng/mL was converted into nmol/L by multiplying by 2.5. We grouped the supplementation duration by allowing a 7 days fluctuation (±7 days) within the same group. We also grouped doses by allowing a 20% fluctuation (±20% IU) of a given dose.

### Statistical analysis

All NMA analyses were performed using the “getmc” package in R version 4.0.4 and the “mvmeta” package in STATA version 13.1. A value of *p* of less than 0.05 was considered statistically significant. A Bayesian NMA was conducted to synthesize direct and indirect comparisons of vitamin D supplementations for improving circulating 25(OH)D level (20). Pooled estimates were reported as MDs with 95%CIs using the Markov Chains Monte Carlo method. The Surface under the cumulative ranking curve (SUCRA) was used to rank the efficacy of treatments, which was expressed as a percentage (21). A higher value indicated a favorable option.

Transitivity, consistency, and homogeneity assumptions had to be satisfied when performing the NMA approach. Transitivity assumption requires the distributions of baseline characteristics (e.g., mean age, the proportion of males, and baseline vitamin D deficiency) or the effect of the control group in different treatment arms to be similar enough to provide valid indirect inferences. Transitivity was presented by violin plots where the overlaps in the y-axis dimension indicated a similarity of characteristics. The consistency assumption requires agreement between direct and indirect evidence. Inconsistency was tested by the node-splitting method, which separated results into direct and indirect evidence from particular comparisons (22). *I^2^* was calculated to assess the heterogeneity, where *I^2^* greater than 50% indicated the existence of heterogeneity, and subgroup analyses were conducted to explore potential factors leading to heterogeneity.

Several subgroup analyses were performed to test whether the results were affected by mean age (≥60 and < 60 years), the proportion of males (≥50 and < 50%), geographic location (Asia, Europe, America, Africa, and Oceania), latitude (≥60^o^, 30^o^-60^o^ and < 30^o^), the detection method of 25(OH)D concentration (LC–MS, ELISA, chemiluminescence, radioimmunoassay, and others), participants with any disease at baseline (yes and no), vitamin D deficiency (yes and no), and co-supplementation with calcium (yes and no). Articles that did not report data on a subgroup basis were classified as an unclear group. Furthermore, publication bias was evaluated by visualization of funnel plots for each comparison.

To recommend the optimum frequency and dosage of vitamin D supplementation for achieving 25(OH)D concentrations greater than 75 nmol/L, we performed meta-regression analyses based on at least 10 studies in each analysis with fixed frequency and dose (23). The analyses aimed to predict follow-up 25(OH)D levels based on baseline 25(OH)D levels. Covariates such as age and disease status did not show significant effects when entered into the meta-regression models and were therefore excluded from the final models. Follow-up 25(OH)D levels at different supplementation frequencies and dosages were estimated using the equations from the random-effects meta-regression models in an inverse manner.

## Results

### Characteristics of included studies

**Figure 1:**
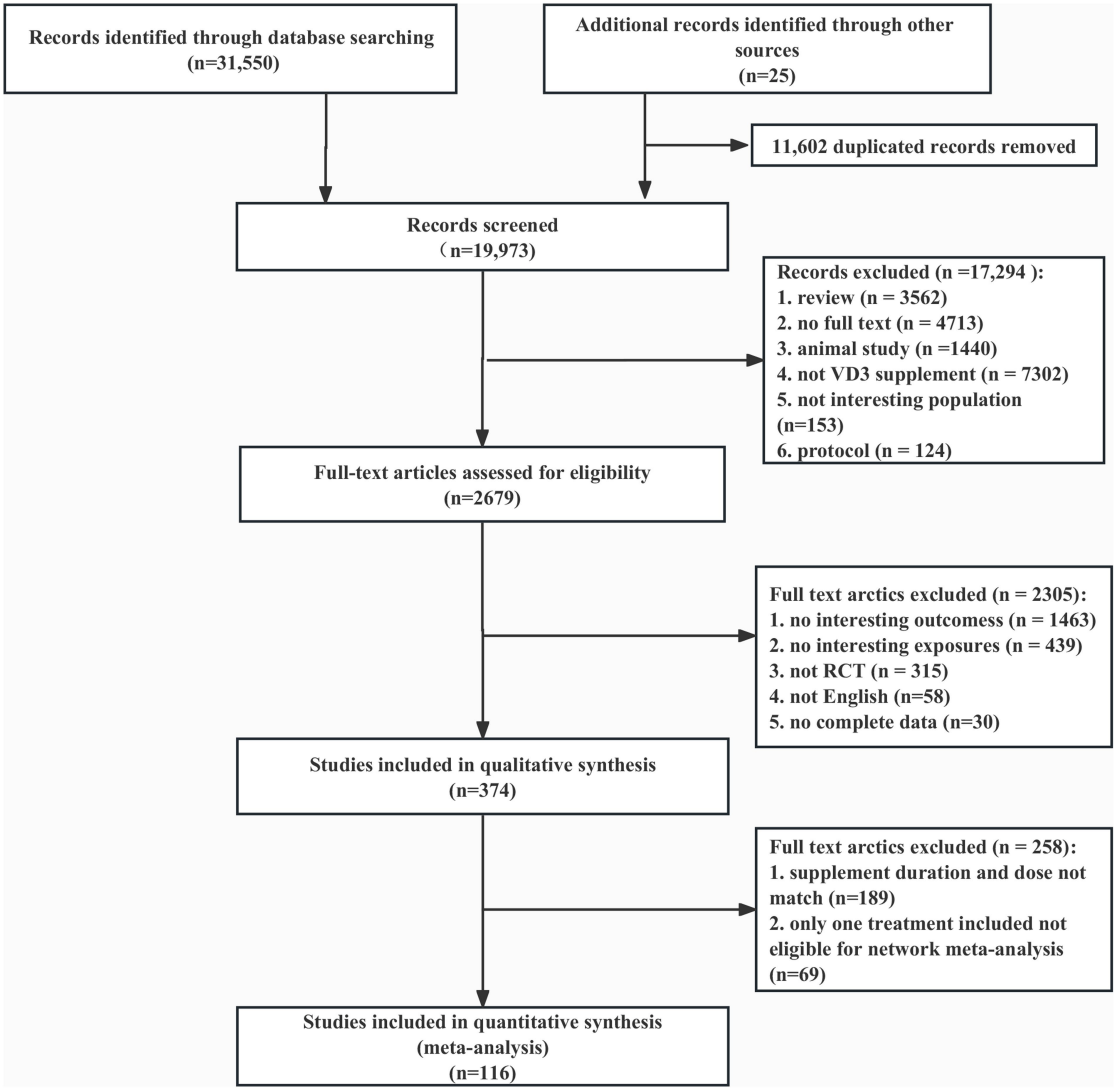
Flowchart of the study selection.

The flowchart of the search strategy is presented in Figure 1. Initially, a total of 31,550 studies were identified from three databases, with 19,973 studies remaining after removing duplicates. Next, 17,294 articles were excluded after scanning titles and abstracts, leaving 2,679 articles for full-text review. Of these, 2,305 studies were excluded due to irrelevant exposure or outcome. Furthermore, 374 potential studies remained, with 258 studies being excluded due to unmatched total vitamin D supplementation doses and durations. Ultimately, 116 RCTs involving 11,376 participants were analyzed in this NMA. The general features of the 116 studies in the NMA are summarized in Table 1; Supplementary Table S3. The vitamin D supplementation dosage ranged from 400 IU to 8,000 IU daily, from 5,000 IU to 50,000 IU weekly, and from 18,000 IU to 120,000 IU monthly. The shortest vitamin D supplementation duration was 2 months, while the longest duration was 12 months. The majority of the trials enrolled participants who were, on average, less than 60 years old, were women, and lived in middle-latitude regions. The proportion of trials including participants with vitamin D deficiency at baseline [25(OH)D concentration of less than 50 nmol/L], taking co-supplementation with calcium, and having any disease at baseline was 26, 5, and 69%, respectively. The most commonly applied detection method for 25(OH)D levels was enzyme-linked immunosorbent assay (ELISA) (31%), followed by chemiluminescence (20%) and liquid chromatography-mass spectrometry (LC–MS) (19%), while radioimmunoassay (8%) was the least used detection method. Regarding the detection method of 25(OH)D levels, 11 (10%)of all trials did not specify the analytical method.

**Table 1:** Characteristics of included studies (*N* = 116).

Variables		*N* (%)
Mean age	<60 years	86 (74)
	≥60 years	27 (23)
	Unclear	3 (3)
Male proportion	<50%	77 (66)
	≥50%	34 (29)
	Unclear	5 (4)
Location	Asia	56 (48)
	Europe	25 (22)
	America	18 (16)
	Oceania	5 (4)
	Africa	3 (3)
	Unclear	9 (8)
Latitude	<30^o^	14 (12)
	30^o^- 59^o^	89 (77)
	≥60^o^	6 (5)
	Unclear	7 (6)
Co-supplementation with Calcium	Yes	6 (5)
	No	110 (95)
Detection method of 25(OH)D	LC–MS	22 (19)
	ELISA	36 (31)
	Chemiluminescence	23 (20)
	Radioimmunoassay	9 (8)
	Others	15 (13)
	Unclear	11 (10)
Participants having any disease at baseline	No	29 (25)
	Yes	80 (69)
	Unclear	7 (6)
Baseline vitamin D deficiency	No	86 (74)
	Yes	30 (26)

LC–MS indicates liquid chromatography-mass spectrometry; ELISA indicates enzyme-linked immunosorbent assay.

### Risk of bias

**Figure 2:**
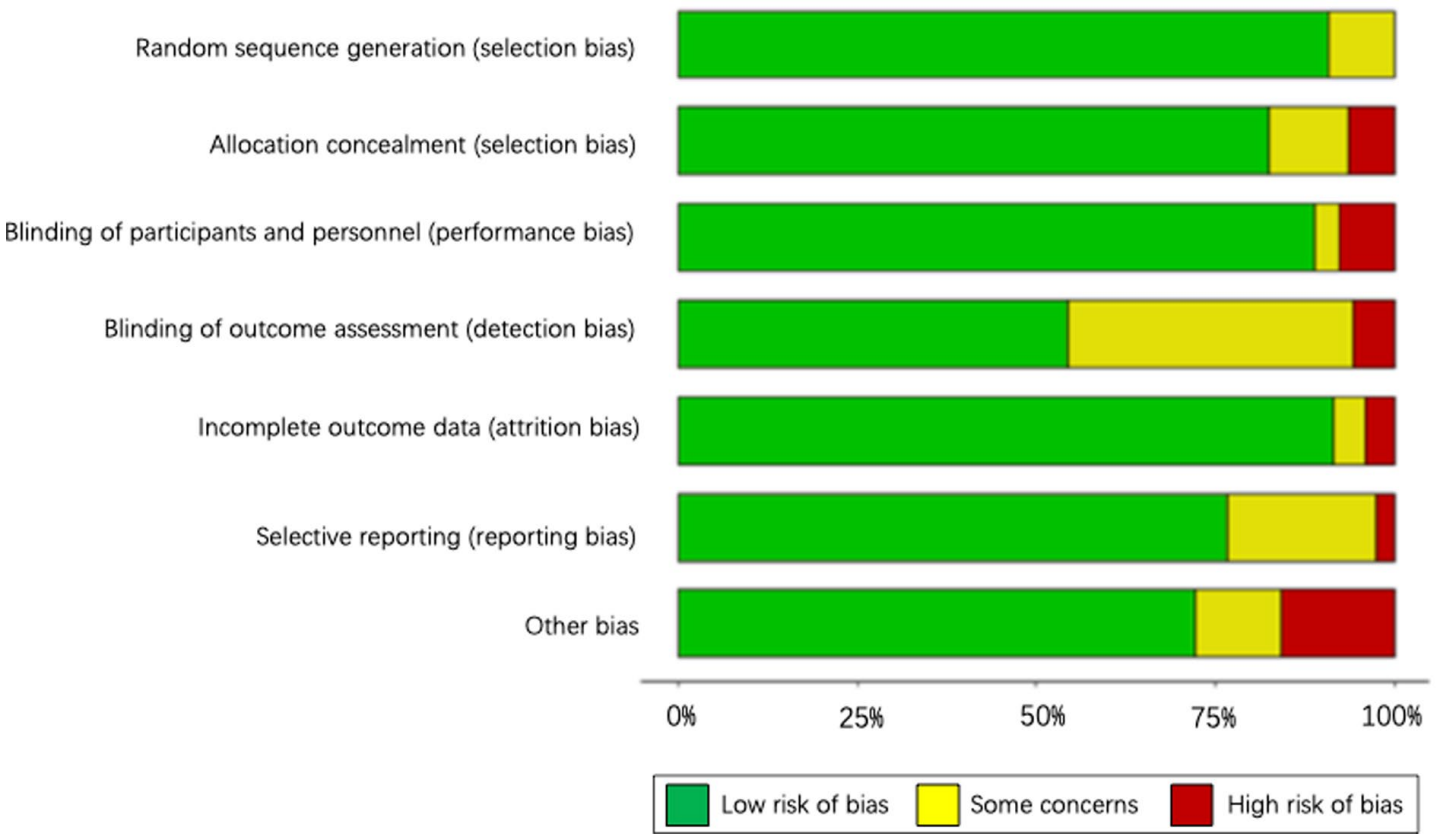
Assessment of the risk of bias using the RoB2 tool.

The assessment of the risk of bias is shown in Figure 2, and the quality of the trials was generally satisfactory. Four domains were at low risk of bias, including random sequence generation (86%), incomplete outcome data (86%), blinding participants and personnel (85%), and allocation concealment (76%). However, an unclear risk of bias was reported in the other three domains, containing blinding of outcome assessment (43%), selective reporting (28%), and other bias (17%). The detailed assessment of the risk of bias for each trial is displayed in Supplementary Table S4.

### Efficacy in elevating circulating 25(OH)D concentrations

The network of eligible comparisons is presented in Supplementary Figure S1, and the main findings of NMA are shown in Table 2. Compared with controls, all comparisons of daily supplementation led to a significant 25(OH)D concentration increase, with pooled mean differences ranging from 31 to 74 nmol/L. In terms of 90,000 IU (~ 1,000 IU/day) vitamin D supplementation during 3 months, statistically significant higher improvement in circulating 25(OH)D concentration was observed in the daily supplementation group, compared with the control group, with a pooled mean difference of 31 nmol/L (95%CI: 21, 40). The result of a total of 180,000 IU (~2,000 IU/day) vitamin D supplements during 3 months was similar, where subjects had a higher 25(OH)D level via daily supplementation (Pooled MD = 38 nmol/L, 95%CI: 28, 47).

**Table 2:** The effect of vitamin D supplementation method on 25(OH)D concentration (nmol/L) using network meta-analysis.

Detecting time	Total dose, IU	Average dose, IU/day	Daily		Weekly		Monthly	
			*N*	Pooled MD (95%CI)	*N*	Pooled MD (95%CI)	*N*	Pooled MD (95%CI)
2 Months	**36,000**	600	2	38 (19,62)	–	–	2	19 (0,41)
	**100,000**	1,666	4	33 (15,50)	2	24 (−5,52)	5	28 (13,44)
	**200,000**	3,333	4	34 (4,64)	5	49 (22,75)	–	–
3 Months	**90,000**	1,000	18	31 (21,40)	3	21 (−2,43)	–	–
	**180,000**	2,000	18	38 (28,47)	–	–	4	32 (11,54)
	**300,000**	3,333	11	34 (19,49)	9	34 (18,50)	–	–
	**600,000**	6,666	2	74 (28,120)	22	63 (49,77)	–	–
6 Months	**300,000**	1,666	6	33 (23,44)	–	–	4	25 (13,38)
	**600,000**	3,333	2	54 (29,79)	13	43 (34,53)	–	–
12 Months	**720,000**	2,000	6	41 (32,51)	–	–	4	24 (13,34)

*N* indicates the number of trials. MD indicates a mean difference. The number in bold indicates its value of *p* is less than 0.05.

Increased effects on 25(OH)D concentrations were reported in most subgroups of intermittent vitamin D supplementation. For example, a significant increase in 25(OH)D levels was noted (Pooled MD = 63 nmol/L, 95%CI: 49, 77) with weekly supplementation of 600,000 IU vitamin D supplements during 3 months (~ 6,666 IU/day) compared to control. However, supplementation of a total of 100,000 IU (~ 1,666 IU/day) during 2 months and a total of 90,000 IU during 3 months (~ 1,000 IU/day) weekly vitamin D supplementation did not affect serum 25(OH)D levels.

In addition, significant pooled mean differences in 25(OH)D levels were observed with monthly vitamin D supplementation for 2 months with a total of 100,000 IU (~ 1,666 IU/day), 3 months with a total of 180,000 IU (~2,000 IU/day), 6 months with a total of 300,000 IU (~1,666 IU/day), and 12 months with a total of 720,000 IU (~2,000 IU/day). However, with a total of 36,000 IU (~ 600 IU/day) during 2 months, monthly vitamin D supplementation had no significant improvements in 25(OH)D levels. SUCRA values are summarized in Table 3 and Table 4 presents the efficacy comparisons between intermittent supplementation and daily supplementation of vitamin D. In most comparisons, although SUCRA values of daily supplementation were higher than those for intermittent supplementation, no significant pooled mean difference between daily supplementation and intermittent supplementation was observed, as their associated 95% CIs included zero. However, daily supplementation was more efficient in elevating 25(OH)D concentrations than monthly supplementation, with a total of 720,000 IU vitamin D supplementation during 12 months (~2,000 IU/day), where the pooled mean difference was 18 nmol/L with a corresponding 95% CI from 4 to 32. Additionally, the effects of the dose and duration of vitamin D supplementation were analyzed (Supplementary Figure S2). Under the given vitamin D supplementation frequency, higher dosage resulted in better efficacy in elevating 25(OH)D levels. However, no dose–response effect was observed for supplementation duration, especially for weekly supplementation.

**Table 3:** The surface under the cumulative ranking curve (SUCRA, %) for all comparisons.

Detecting time	Total dose, IU	Average dose, IU/day	Daily	Weekly	Monthly
2 Months	**36,000**	600	100	–	50
	**100,000**	1,666	83	52	64
	**200,000**	3,333	58	92	–
3 Months	**90,000**	1,000	92	57	–
	**180,000**	2,000	85	–	65
	**300,000**	3,333	75	75	–
	**600,000**	6,666	85	65	–
6 Months	**300,000**	1,666	94	–	56
	**600,000**	3,333	91	59	–
12 Months	**720,000**	2,000	100	–	50

**Table 4:** The effect of intermittent vitamin D supplementation method on 25(OH)D concentration (nmol/L), compared to daily supplementation using network meta-analysis.

Detecting	Total dose	Average dose	Weekly		Monthly	
Time	IU	IU/day	*N*	Pooled MD (95%CI)	*N*	Pooled MD (95%CI)
2 Months	**36,000**	600	–	–	2	−19 (−45,5)
	**100,000**	1,666	2	−9 (−38,21)	5	−5 (−26,17)
	**200,000**	3,333	5	15 (−26,55)	–	–
3 Months	**90,000**	1,000	3	−10 (−34,13)	–	–
	**180,000**	2,000	–	–	4	−6 (−29,19)
	**300,000**	3,333	9	0 (−22,22)	–	–
	**600,000**	6,666	22	−11 (−59,37)	–	–
6 Months	**300,000**	1,666	–	–	4	−8 (−25, 8)
	**600,000**	3,333	13	−11 (−37,16)	–	–
12 Months	**720,000**	2,000	–	–	4	−18 (−32, −4)

MD indicates mean difference. Number in bold indicates its p value less than 0.05.

Furthermore, we combined similar doses into one group under each supplementation frequency to recommend the optimal vitamin D supplementation frequency and dose by inverse using random-effects meta-regression models (Table 5). When the analysis was conducted among the group with more than 10 trials (to ensure statistical power), an average of more than 2,000 IU vitamin D supplementation per day could raise 25(OH)D levels higher than 75 nmol/L. The efficacy of elevating 25(OH)D level was similar between 2,000 IU daily and 60,000 IU monthly (~2,000 IU/day) vitamin D supplementation on (87 vs. 85 nmol/L).

**Table 5:** Associations of vitamin D supplementation and achieved 25(OH)D concentrations (nmol/L) using random-effects meta-regression models.

Variables		N*N*	Beta (95%CI)	*R* ^2^	Achieved 25(OH)D concentration, nmol/L
Daily 1,000 IU	Vitamin D supplementation	19	28 (23,33)	0.88	70
	Baseline 25(OH)D concentration, nmol/L		1 (0.8,1.2)		
Daily 2,000 IU	Vitamin D supplementation	33	37 (32,42)	0.85	87
	Baseline 25(OH)D concentration, nmol/L		0.9 (0.7,1.1)		
Daily 3,000 IU	Vitamin D supplementation	16	40 (29,51)	0.75	84
	Baseline 25(OH)D concentration, nmol/L		1.2 (0.8,1.7)		
Weekly 25,000 IU	Vitamin D supplementation	22	43 (33,53)	0.75	91
	Baseline 25(OH)D concentration, nmol/L		1.3 (0.9,1.7)		
Weekly 50,000 IU	Vitamin D supplementation	22	64 (50,77)	0.71	107
	Baseline 25(OH)D concentration, nmol/L		1.1 (0.6,1.7)		
Monthly 60,000 IU	Vitamin D supplementation	15	31 (20,42)	0.85	85
	Baseline 25(OH)D concentration, nmol/L		0.9 (0.7,1.0)		

*N* indicates the number of trials. *R*^2^ indicates the *R* square of meta-regression models.

### Consistency, transitivity, and heterogeneity

The mean age, male proportion, sample size, and baseline serum 25(OH)D levels showed similar variation ranges across intervention comparisons, indicating that the transitivity assumption was not violated (Supplementary Figure S3). Random consistency models were used due to their lower DICs, and there was no global inconsistency for any of the comparisons (all *p* > 0.05). The node-split model was used to evaluate the local inconsistency, and no significant disparities were observed between the direct and indirect comparisons (Supplementary Figure S4). However, high heterogeneity (>50%) was noted for all comparisons except for a total of 36,000 IU vitamin D supplementation during 2 months (*I^2^* = 6%). Comparison-adjusted funnel plots, which are presented in Supplementary Figure S5, indicated no significant risk of small-study effect.

### Sensitivity analysis

Due to the high heterogeneity, we conducted subgroup analyses stratified by potential confounding factors such as mean age, male proportion, latitude, geographic location, co-supplementation with calcium, the detection method of 25(OH)D level, and participants with any disease or vitamin D deficiency at baseline (Supplementary Tables S5 – S13). For instance, when taking a total of 300,000 IU vitamin D supplementation during 3 months as an example, significant increases in serum 25(OH)D levels were observed by daily supplementation in subgroups with participants under 60 years of age, residing in middle latitude regions, living in Asia, Europe, and America, those without co-supplementation with calcium, and no vitamin D deficiency at baseline compared to the control group. However, the subgroup results stratified by male proportion and disease status at baseline were consistent with the main results. As for weekly supplementation, significant pooled MDs were also found in subgroups of detection method by ELISA and LC–MS, and participants with any disease at baseline. In addition, the subgroup results for a total of 36,000 IU vitamin D supplementation during 2 months were not shown due to the limited number of included trials (*n* = 2).

## Discussion

This network meta-analysis included 116 trials and demonstrated that vitamin D supplementation elevated the circulating 25(OH)D level without a significant difference in efficacy between daily and intermittent supplementation with similar dosages. Additionally, individuals should intake at least 60,000 IU of vitamin D supplements monthly (~2,000 IU/day) to achieve sufficient 25(OH)D concentrations.

Our previous meta-analysis (5) also reported that vitamin D supplementation significantly elevated the 25(OH)D level in subjects. Athletes with vitamin D insufficiency who received 3,000 IU of daily vitamin D supplementation experienced a significant rise in 25(OH)D concentration (MD = 15.2 ng/mL, 95%CI: 10.7–19.7) (24). In elderly individuals (age > 60 years), pooling results from eight RCTs, including 1,293 participants, revealed an elevated serum 25(OH)D level in the vitamin D supplementation group compared with the control group (MD = 13.84 ng/mL, 95%CI: 10.21–17.47) (25). These findings align with the results of this NMA meta-analysis, which demonstrated that vitamin D supplementation substantially improves circulating 25(OH)D levels. To date, there has been no integrated evidence comparing the efficacy of 25(OH)D levels between daily and intermittent vitamin D supplementation. A trial conducted in healthy women showed no significant differences in 25(OH)D concentration at day 28 between 5,000 IU daily vitamin D supplementation for 28 days and a single dose of 150,000 IU vitamin D (130.5 ± 25.1 vs. 122.0 ± 24.8 nmol/L, *p* = 0.28) (26). After giving vitamin D supplements to 64 adult subjects with vitamin D deficiency for 3 months, the study revealed that there was an equivalent effect on serum 25(OH)D levels across 1,000 IU daily (MD = 13.0 ng/mL), 7,000 IU weekly (MD = 12.6 ng/mL) and 30,000 IU monthly vitamin D supplementation (MD = 12.9 ng/mL) (27). Moreover, no significant differences in serum 25(OH)D concentrations were noted in elderly patients with hip fractures among 1,500 IU daily (MD = 33.2 ng/mL), 10,500 IU weekly (MD = 29.2 ng/mL), or 45,000 IU monthly (MD = 37.1 ng/mL) vitamin D supplementation for 2 months (28). Our findings are consistent with these trials, implying that there was equal efficacy of circulating 25(OH)D concentration between daily and intermittent vitamin D supplementation.

Furthermore, 60,000 IU monthly (~2,000 IU/day) vitamin D supplementation was suggested to reach optimal 25(OH)D concentration, which was coherent with the recommendation given for adults by the United States Endocrine Society (1,500–2,000 IU per day) and our previous meta-analysis (2,214 IU per day) (4, 5). A study conducted by Meekins et al. found that intermittent supplementation resulted in a rapid increase in 25(OH)D concentration during the first week, followed by a slow decline; meanwhile, daily supplementation resulted in a gradual rise in 25(OH)D concentration, reaching a plateau at 1 month or so (26). No significant difference in 25(OH)D level was demonstrated at the endpoint between the intermittent and daily groups, but the area under the curve of the intermittent group was greater. This indicates that the body maintained a higher 25(OH)D level by intermittent supplementation, which indirectly suggests a greater benefit of intermittent supplementation.

It is worth noting that this NMA has several strengths. We included 116 RCTs with a total sample size of 11,376 subjects and assessed 10 combinations of dose and duration, which allowed us to comprehensively evaluate the efficacy of vitamin D supplementation strategies in elevating 25(OH)D concentration. However, we could not rule out some limitations. First, high heterogeneity was observed due to the large number of studies that were included. However, subgroup analyses based on mean age, male proportion, 25(OH)D detection method, latitude, geographic location, participants’ disease status at baseline, and baseline vitamin D partially explained part of the heterogeneity. Since different 25(OH)D detection methods may lead to a variation of 25(OH)D levels, we excluded studies with unknown detection methods and repeated the main analysis. The finding did not significantly alter. It should be noted that the number of trials in subgroups with unconfirmed results was small, with only one or two trials included. In addition to heterogeneity, another reason for unstable results was the limited power to detect significant pooled mean differences with a small number of studies in some subgroups. Second, multiple comparisons were made, which could increase the risk of making a type I error. When making indirect comparisons, we were subject to the influence of confounding factors and had low power to test for inconsistency. Moreover, some trials were excluded due to unmatched total doses and durations of vitamin D supplementation, which could induce some publication bias. However, the funnel plot did not suggest any apparent publication bias. It should be noted that the general characteristics, such as mean age, and male proportion between included and excluded studies, were comparable. Finally, the number of trials in some situations was small; for example, only four trials were included in the final analysis of a total of 36,000 IU of vitamin D supplementation over 2 months. Nevertheless, the results across multiple combinations were consistent.

In conclusion, our analysis suggests that intermittent and daily vitamin D supplementation have similar efficacy in improving circulating 25(OH)D levels under equivalent cumulative dosage and duration. For achieving a sufficient 25(OH)D concentration, we recommend 60,000 IU monthly vitamin D supplementation due to its convenience and efficiency.

## Data availability statement

The original contributions presented in the study are included in the article/Supplementary material, further inquiries can be directed to the corresponding authors.

## Author contributions

YZ: data curation, data collection and extraction, formal analysis and interpretation of data, and writing-original draft preparation. ZZ: data collection and extraction, formal analysis and interpretation of data, and writing-original draft preparation. PC and HZ: data collection and extraction, and formal analysis. ZP, HC, XX, and WL: data collection and extraction. SS and MM: writing-reviewing and editing. DC: funding acquisition, and writing-reviewing and editing. HL: conceptualization and design, interpretation of data, funding acquisition, and writing-reviewing and editing. YY: conceptualization and design, funding acquisition, and writing-reviewing and editing. All authors have read and approved the manuscript.

## Funding

This study was funded by the National Natural Science Foundation of China (81973055, 82103807, and 81873839), major research and development projects of the Zhejiang Science and Technology Department (2018C03010), and the Fundamental Research Funds for the Central Universities.

## Conflict of interest

The authors declare that the research was conducted in the absence of any commercial or financial relationships that could be construed as a potential conflict of interest.

## Publisher’s note

All claims expressed in this article are solely those of the authors and do not necessarily represent those of their affiliated organizations, or those of the publisher, the editors and the reviewers. Any product that may be evaluated in this article, or claim that may be made by its manufacturer, is not guaranteed or endorsed by the publisher.

## Abbreviations

25(OH)D: 25-hydroxyvitamin D
CI: confidence interval
ELISA: enzyme-linked immunosorbent assay
LC–MS: liquid chromatography-mass spectrometry
MD: mean difference
NMA: network meta-analysis
PRISMA: Preferred Reporting Items for Systematic Reviews and Meta-analyses
RCT: randomized controlled trials
RoB: Risk of bias
SUCRA: Surface under the cumulative ranking curve
