# Supplementary material for: Efficacy of intermittent versus daily vitamin D supplementation on improving circulating 25(OH)D concentration: a Bayesian network meta-analysis of randomized controlled trials
Source: Front Nutr. 2023 Aug 24;10:1168115. doi: 10.3389/fnut.2023.1168115 (PMC10488712; doi:10.3389/fnut.2023.1168115)
Supplement: Supplementary file 1 [file Table_1.DOCX]

**A B**

**
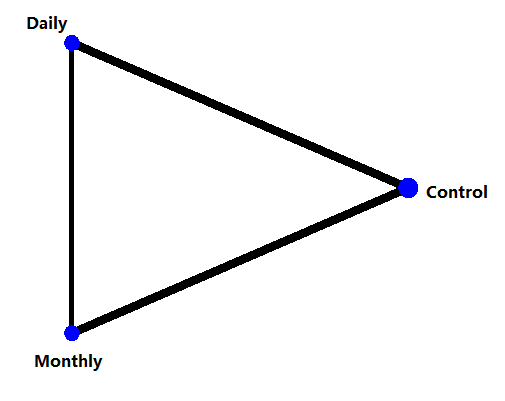
**
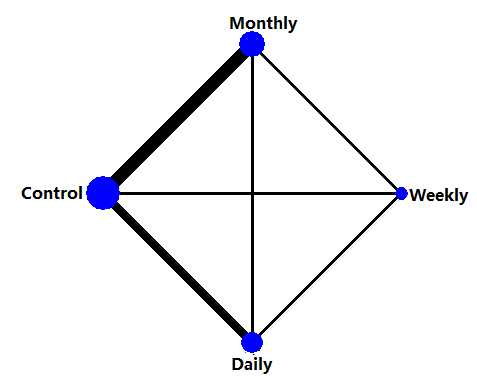


**C D**

**
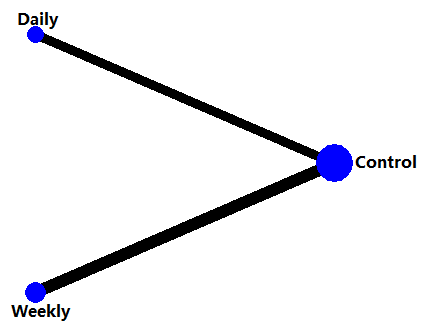

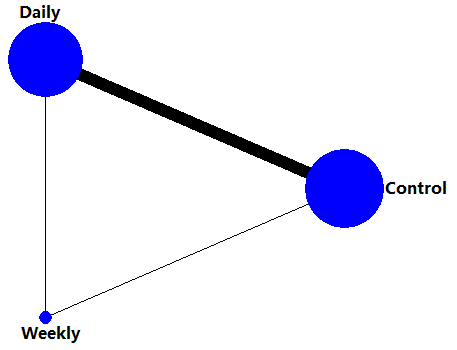
**

**E F**

**
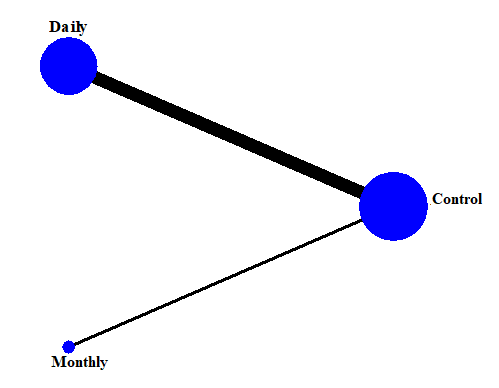

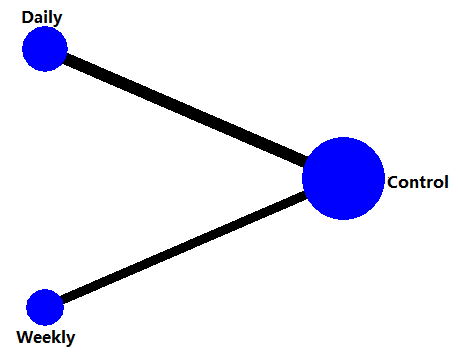
**

**G H**

**
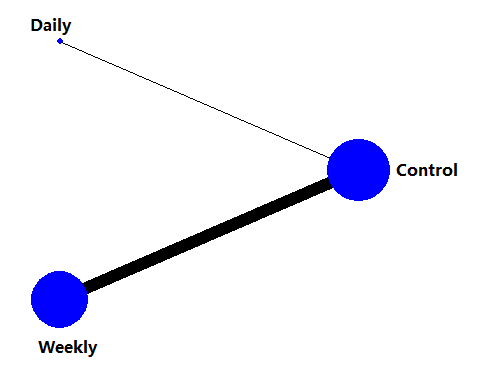

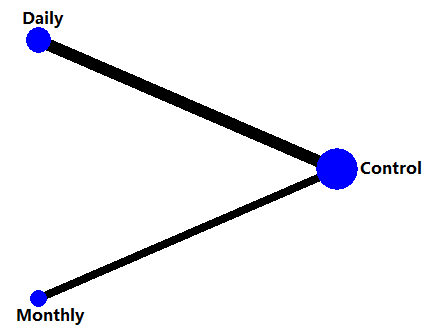
**

**I J**

**
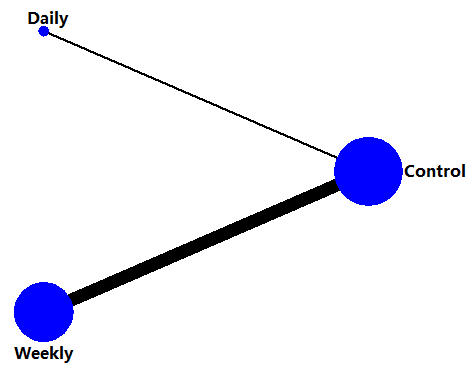

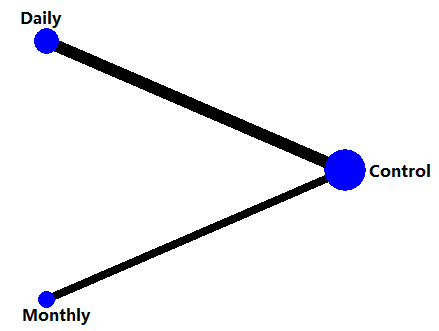
**

**Figure S1.** **Network of available comparisons between different vitamin D supplementation method on 25(OH)D concentration (nmol/L).** A. Total 36,000 IU vitamin D supplementation during two months; B. Total 100,000 IU vitamin D supplementation during two months; C. Total 200,000 IU vitamin D supplementation during two months; D. Total 90,000 IU vitamin D supplementation during three months; E. Total 180,000 IU vitamin D supplementation during three months; F. Total 300,000 IU vitamin D supplementation during three months; G. Total 600,000 IU vitamin D supplementation during three months; H. Total 300,000 IU vitamin D supplementation during six months; I. Total 600,000 IU vitamin D supplementation during six months; J. Total 720,000 IU vitamin D supplementation during twelve months. Size of node is proportional to number of trial participants, and thickness of continuous line connecting nodes is proportional to number of participants randomized in trials directly comparing the two treatments.
