# Supplementary material for: Efficacy of intermittent versus daily vitamin D supplementation on improving circulating 25(OH)D concentration: a Bayesian network meta-analysis of randomized controlled trials
Source: Front Nutr. 2023 Aug 24;10:1168115. doi: 10.3389/fnut.2023.1168115 (PMC10488712; doi:10.3389/fnut.2023.1168115)
Supplement: Supplementary file 2 [file Table_2.DOCX]

**A**

**B**

**C**

**D**

**E**

**F**

**Figure S2.** **The effect of duration time and dose on 25(OH)D concentration (nmol/L) under the same supplementation way.** A. Dose effect for daily supplementation; B. Dose effect for weekly supplementation; C. Dose effect for monthly supplementation; D. Duration effect for daily supplementation; E. Duration effect for weekly supplementation; F. Duration effect for monthly supplementation.
