# Supplementary material for: Efficacy of intermittent versus daily vitamin D supplementation on improving circulating 25(OH)D concentration: a Bayesian network meta-analysis of randomized controlled trials
Source: Front Nutr. 2023 Aug 24;10:1168115. doi: 10.3389/fnut.2023.1168115 (PMC10488712; doi:10.3389/fnut.2023.1168115)
Supplement: Supplementary file 3 [file Table_3.DOCX]

**A**


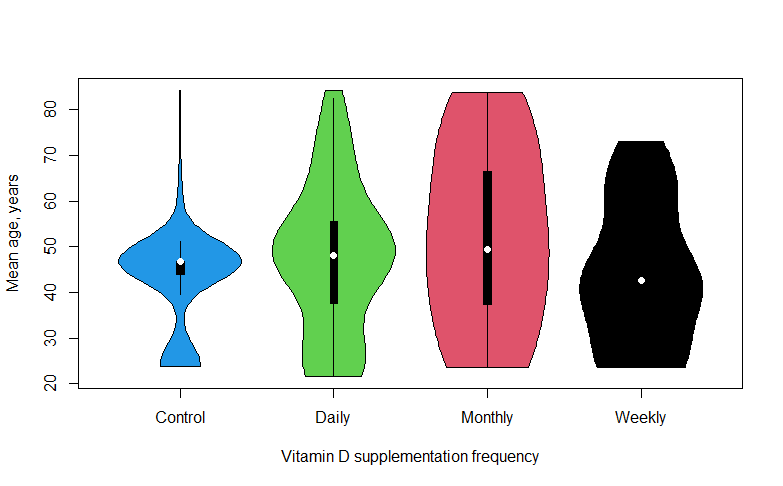


**B**


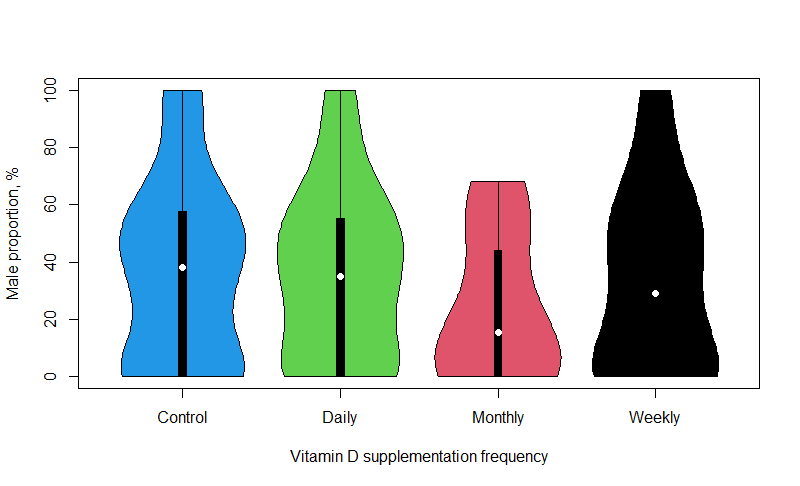


**C**


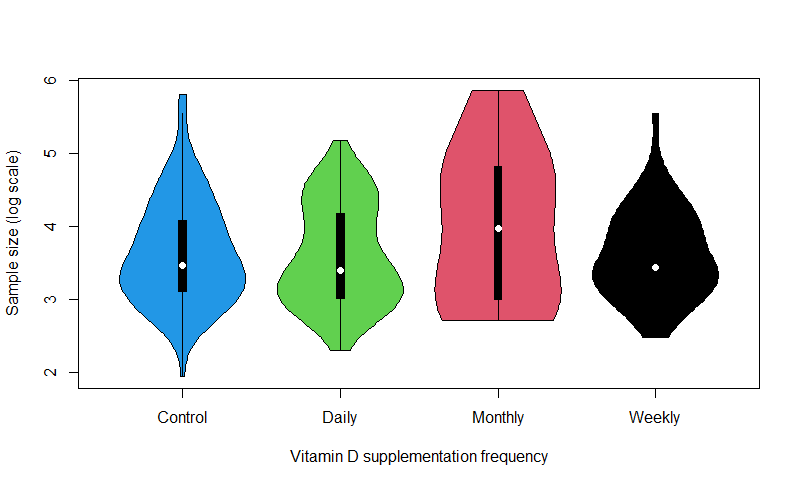


**D**


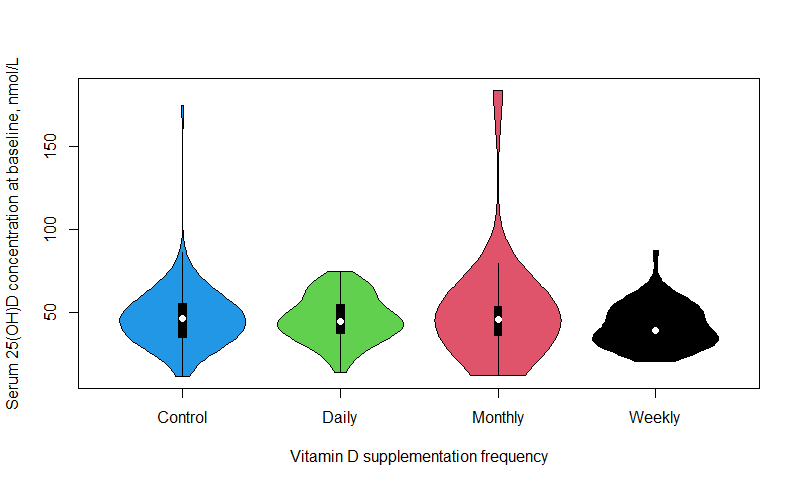


**Figure S3. Assessing transitivity between different vitamin D supplementation frequency using violin plot.** A. Distribution of mean age; B. Distribution of male proportion; C. Distribution of sample size in log scale; D. Distribution of serum 25(OH)D concentration at baseline (nmol/L). The overlap of the y-axis dimension indicates the similar distribution of characteristics.
