# Supplementary material for: Efficacy of intermittent versus daily vitamin D supplementation on improving circulating 25(OH)D concentration: a Bayesian network meta-analysis of randomized controlled trials
Source: Front Nutr. 2023 Aug 24;10:1168115. doi: 10.3389/fnut.2023.1168115 (PMC10488712; doi:10.3389/fnut.2023.1168115)
Supplement: Supplementary file 4 [file Table_4.DOCX]

**A**

**
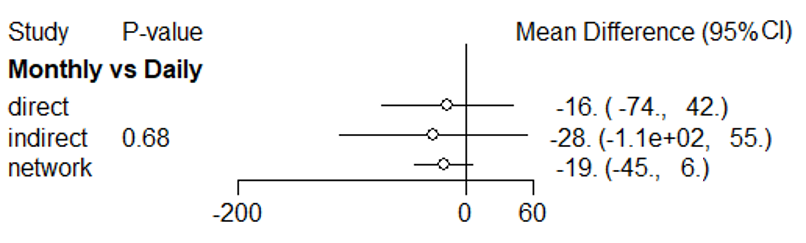
**

**B**

**
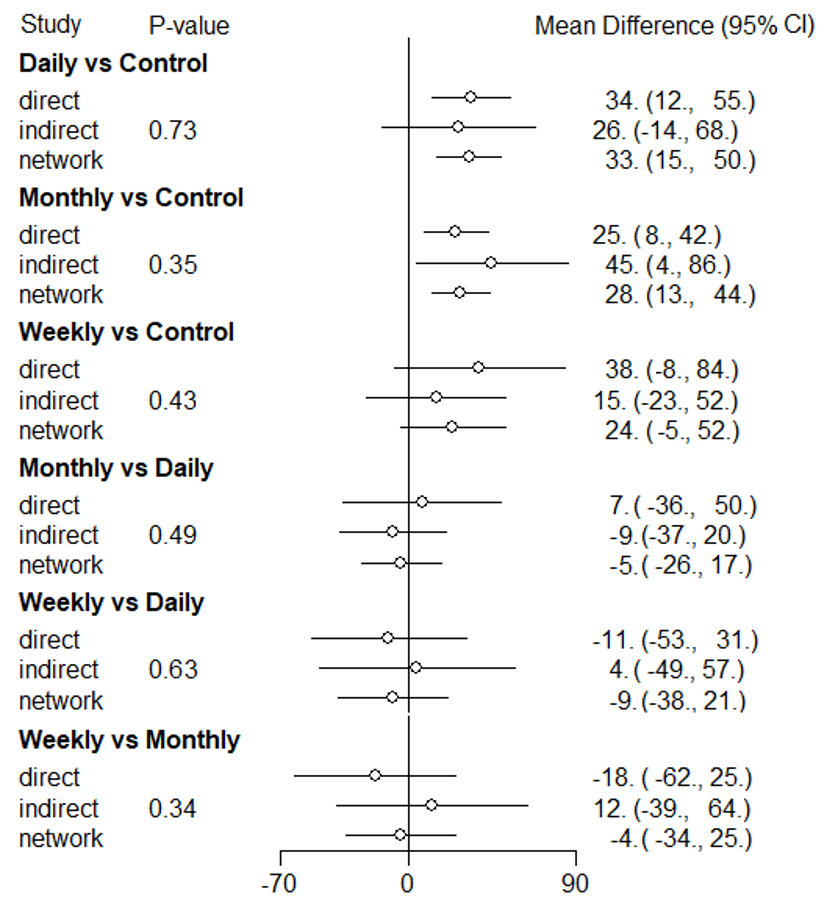
**

**C**

**
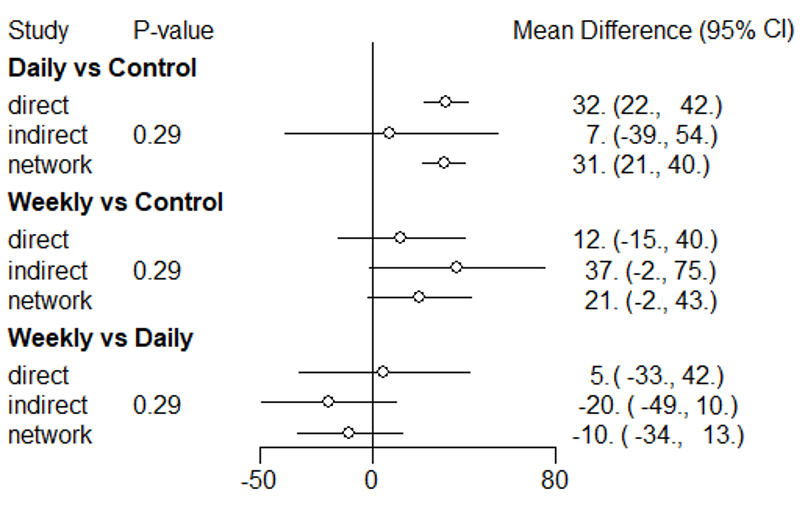
**

**Figure S4.** **Assessing local inconsistency for improving 25(OH)D concentration (nmol/L) using node-split model**. A. Total 36,000 IU vitamin D supplementation during two months; B. Total 100,000 IU vitamin D supplementation during two months; C. Total 90,000 IU vitamin D supplementation during three months.
