# Supplementary material for: Efficacy of intermittent versus daily vitamin D supplementation on improving circulating 25(OH)D concentration: a Bayesian network meta-analysis of randomized controlled trials
Source: Front Nutr. 2023 Aug 24;10:1168115. doi: 10.3389/fnut.2023.1168115 (PMC10488712; doi:10.3389/fnut.2023.1168115)
Supplement: Supplementary file 5 [file Table_5.DOCX]

**A**

**B**

**C**

**D**

**E**

**F**

**G**

**H**

**I**

**J**

**Figure S5.** **Funnel plot for comparison-specific pooled mean differences.** A. Total 36,000 IU vitamin D supplementation during two months; B. Total 100,000 IU vitamin D supplementation during two months; C. Total 200,000 IU vitamin D supplementation during two months; D. Total 90,000 IU vitamin D supplementation during three months; E. Total 180,000 IU vitamin D supplementation during three months; F. Total 300,000 IU vitamin D supplementation during three months; G. Total 600,000 IU vitamin D supplementation during three months; H. Total 300,000 IU vitamin D supplementation during six months; I. Total 600,000 IU vitamin D supplementation during six months; J. Total 720,000 IU vitamin D supplementation during twelve months.
