# Supplementary material for: Efficacy of intermittent versus daily vitamin D supplementation on improving circulating 25(OH)D concentration: a Bayesian network meta-analysis of randomized controlled trials
Source: Front Nutr. 2023 Aug 24;10:1168115. doi: 10.3389/fnut.2023.1168115 (PMC10488712; doi:10.3389/fnut.2023.1168115)
Supplement: Supplementary file 7 [file Table_7.DOCX]

| Table S2. Search strategy. | |
| --- | --- |
| **Database** | **Search strategy** |
| **MEDLINE** | 1. 1. “Vitamin D”[Mesh] OR vitamin D OR “Cholecalciferol”[Mesh] OR “Cholecalciferols” OR “Vitamin D3” OR vitamin D 3 OR colecalciferol 2. 2. "randomized controlled trial"[pt] OR "controlled clinical trial"[pt] OR randomized[tiab] OR trial[tiab] 3. 3. "Humans"[MeSH Terms] OR human[tiab] OR volunteer*[tiab] OR participant*[tiab] OR subject*[tiab] OR people [tiab] 4. 4. #1 AND #2 AND #3 |
| **EMBASE** | 1. 1. 'vitamin d'/exp OR 'vitamin d' OR 'cholecalciferol'/exp OR 'cholecalciferol' OR  'colecalciferol'/exp OR 'colecalciferol' OR 'vitamin d3'/exp OR 'vitamin d3' OR 'vitamin d 3' 2. 2. (randomized controlled trial):pt OR (controlled clinical trial):pt OR (randomized):ti,ab,kw OR (trial):ti,ab,kw 3. 3. ‘Human’/exp OR human:ti,ab,kw OR volunteer*:ti,ab,kw OR participant*:ti,ab,kw OR subject*:ti,ab,kw OR people:ti,ab,kw 4. 4. #1 AND #2 AND #3 |
| **Cochrane library** | 1. 1. [mh “Vitamin D”] OR Vitamin-D OR Cholecalciferol* OR Vitamin-D3 OR vitamin-D-3 OR colecalciferol 2. 2. (randomized controlled trial):pt OR (controlled clinical trial):pt OR (randomized):ti,ab,kw OR (trial):ti,ab,kw 3. 3. [mh human] OR human:ti,ab,kw OR volunteer*:ti,ab,kw OR participant*:ti,ab,kw OR subject*:ti,ab,kw OR people:ti,ab,kw 4. 4. #1 AND #2 AND #3 |
