# Supplementary material for: Efficacy of intermittent versus daily vitamin D supplementation on improving circulating 25(OH)D concentration: a Bayesian network meta-analysis of randomized controlled trials
Source: Front Nutr. 2023 Aug 24;10:1168115. doi: 10.3389/fnut.2023.1168115 (PMC10488712; doi:10.3389/fnut.2023.1168115)
Supplement: Supplementary file 8 [file Table_8.DOCX]

| Table S3. General information of included studies (N=116). | | | | | | | | | |
| --- | --- | --- | --- | --- | --- | --- | --- | --- | --- |
| Study | Region | Population | Vitamin D deficiency | Mean age, years* | male%* | Sample size | Intervention | Serum VD detection method | Calcium co-supplementation |
| Abdollahi et al.  (2019)[1] | NA | Diseased population | Yes | 23/23 | 0/0 | 130 | 2000 IU daily vs. placebo: supplement duration is 12 weeks | ELISA | No |
| Agarwal et al.  (2013)[2] | India | General population | No | 54/54/56 | 0/0/0 | 64 | 500 IU/1000 IU daily vs. placebo: supplement duration is 3 months | Radioimmunoassay | Yes |
| Al-Bayyari et al. (2020)[3] | Jordan | Diseased population | Yes | 24/24 | 0/0 | 58 | 50000 IU weekly vs. placebo: supplement duration is 60 days | others | No |
| Aloia et al.  (2015)[4] | USA | General population | No | 60/59/57/60 | 0/0/0/0 | 71 | 800 IU /2000 IU/4000 IU daily vs. placebo: supplement duration is 8 weeks | Radioimmunoassay | No |
| Amani et al.  (2018)[5] | Iran | Diseased population | No | 38/38 | 36/29 | 85 | 50000 IU biweekly vs. placebo: supplement duration is 6 months | Radioimmunoassay | No |
| Ameri et al.  (2013)[6] | Italy | General population | No | 61/63/62 | 31/21/42 | 39 | 5000 IU/7000 IU weekly vs. placebo: supplement duration is 12 weeks | Chemiluminescence | No |
| Amorim et al.  (2017)[7] | Portugal | Diseased population | No | 48/43 | NA/ | 9 | 25000 IU biweekly vs. placebo: supplement duration is 8 weeks | Chemiluminescence | No |
| Anaraki et al.  (2017)[8] | Iran | Diseased population | Yes | 44/44 | 41/30 | 56 | 50000 IU weekly vs. placebo: supplement duration is 12 weeks | ELISA | No |
| Anyanwu et al.  (2016)[9] | Nigeria | Diseased population | No | 53/51 | 44/41 | 33 | 3000 IU daily vs. placebo: supplement duration is 12 weeks | LC-MS | No |
| Ardabili et al.  (2012)[10] | Iran | Diseased population | Yes | 27/27 | 0/0 | 50 | 50000 IU every 20 days vs. placebo: supplement duration is 2 months | Chemiluminescence | No |
| Arjeh, S. et al.  (2020)[11] | Iran | Diseased population | Yes | 31/33 | 0/0 | 55 | 50000 IU weekly vs. placebo: supplement duration is 12 weeks | ELISA | No |
| Ayuso et al.  (2018)[12] | Spain | General population | Yes | 27/27 | 100/100 | 36 | 3000 IU daily vs. placebo: supplement duration is 8 weeks | LC-MS | No |
| Barchetta et al. (2016)[13] | Italy | Diseased population | No | 57/60 | 70/60 | 55 | 2000 IU daily vs. placebo: supplement duration is 24 weeks | NA | No |
| Barnes et al.  (2006)[14] | Northern Island | General population | No | 22/21 | 47/58 | 27 | 600 IU daily vs. placebo: supplement duration is 8 weeks | ELISA | No |
| Bhagatwala et al. (2015)[15] | USA | Diseased population | Yes | 26/24/26/28 | 25/13/18/12 | 65 | 18000 IU/60000 IU/120000 IU monthly vs. placebo: supplement duration is 8 weeks | ELISA | No |
| Bidar et al.  (2012)[16] | Tehran | Diseased population | No | NA | 38/48 | 100 | 500 IU daily vs. placebo: supplement duration is 12 weeks | LC-MS | Yes |
| Brisson et al.  (2017)[17] | Canada | General population | No | 43/43/43/43 | 0/0/0/0 | 391 | 1000 IU/2000 IU/3000 IU daily vs. placebo: supplement duration is 1 year | LC-MS | No |
| Buonfiglio et al. (2017)[18] | USA | General population | No | 26/29 | 71/58 | 40 | 1000 IU daily vs. placebo: supplement duration is 90 days | others | No |
| Carrillo et al.  (2012)[19] | USA | Diseased population | No | 26/26 | 50/46 | 23 | 4000 IU daily vs. placebo: supplement duration is 12 weeks | others | Yes |
| Cavalcante. et al (2015)[20] | NA | Diseased population | Yes | 62/62 | 0/0 | 38 | 6600 IU weekly vs. placebo; supplement duration is 12 weeks | Chemiluminescence | No |
| Chandler et al.  (2015)[21] | USA | General population | No | 51/51/50/51 | 27/34/35/33 | 254 | 1000 IU/2000 IU/4000 IU daily vs. placebo: supplement duration is 3 months | Radioimmunoassay | Yes |
| Chel et al.  (2008)[22] | NA | Diseased population | No | 84/47/84/84 | 24/28/16/26 | 187 | 4200IU/600IU/18000IU monthly vs. placebo: supplement duration is 2 months | Radioimmunoassay | No |
| Cherniack et al. (2011)[23] | USA | General population | No | 80/80 | 98/98 | 34 | 2000 IU daily vs. placebo: supplement duration is 3 months | Radioimmunoassay | No |
| Crew et al.  (2019)[24] | USA | General population | No | 44/45 | 0/0 | 148 | 20000 IU weekly vs. placebo: supplement duration is 6 months | LC-MS | No |
| Dabbaghmanesh et al. (2018)[25] | Iran | Diseased population | Yes | 45/46 | 41/41 | 63 | 50000 IU weekly vs. placebo: supplement duration is 12 weeks | NA | No |
| Dadaei et al.  (2015)[26] | Iran | Diseased population | Yes | 37/39 | 42/49 | 82 | 50000 IU weekly vs. placebo: supplement duration is 12 weeks | ELISA | No |
| Damghanian et al. (2019)[27] | Iran | Diseased population | No | 50/50 | 57/57 | 35 | 4000 IU daily vs. placebo: supplement duration is 2 months | ELISA | No |
| Ebrahimkhani et al. (2020)[28] | Iran | Diseased population | Yes | 51/52 | 82/68 | 36 | 50000 IU weekly vs. placebo: supplement duration is 12 weeks | Chemiluminescence | No |
| El Hajj et al.  (2018)[29] | Lebanese | General population | Yes | 73/74 | 55/47 | 115 | 10000 IU 3 times a week vs. placebo: supplement duration is 6 months | Radioimmunoassay | No |
| El Hajj et al.  (2020)[30] | Lebanon | Diseased population | Yes | 67/47 | 51/51 | 88 | 10000 IU 3 times a week vs. placebo: supplement duration is 6 months | Radioimmunoassay | No |
| Farag et al.  (2018)[31] | Iran | Diseased population | No | 41/43 | 33/52 | 49 | 2000 IU daily vs. placebo: supplement duration is 12 weeks | NA | No |
| Farrokhian et al. (2017)[32] | Iran | Diseased population | Yes | 61/63 | 50/50 | 60 | 50000 IU biweekly vs. placebo: supplement duration is 6 months | ELISA | No |
| Foroozanfard et al. (2017)[33] | Iran | Diseased population | No | 18-40 | 0/100/0 | 90 | 1000 IU/4000 IU daily vs. placebo: supplement duration is 12 weeks | ELISA | No |
| Gao et al.  (2020)[34] | China | Diseased population | No | 49/51/53 | 52/48/49 | 323 | 1000 IU/2000 IU daily vs. placebo: supplement duration is 90 days | ELISA | No |
| Geier et al.  (2018)[35] | Switzerland | Diseased population | No | 39/50 | NA/ | 18 | 2100 IU daily vs. placebo: supplement duration is 48 weeks | others | No |
| Ghaderi et al.  (2017)[36] | Iran | Diseased population | No | 40/43 | NA/ | 68 | 50000 IU biweekly vs. placebo: supplement duration is 12 weeks | ELISA | No |
| Ghaderi et al.  (2020)[37] | Iran | Diseased population | No | 38/41 | 100/100 | 64 | 50000 IU biweekly vs. placebo: supplement duration is 24 weeks | ELISA | No |
| Ghaderi et al.  (2020)[38] | Iran | Diseased population | No | 41/43 | 100/100 | 40 | 50000 IU biweekly vs. placebo: supplement duration is 12 weeks | ELISA | No |
| Ghorbani et al. (2020)[39] | Iran | Diseased population | No | 37/38 | 20/20 | 74 | 2000 IU daily vs. placebo: supplement duration is 12 weeks | Chemiluminescence | No |
| Glendenning et al. (2012)[40] | Australia | General population | No | 77/77 | 0/0 | 40 | 150000 IU quarterly vs. placebo: supplement duration is 3 months | NA | No |
| Goncalves-Mendes et al. (2019)[41] | France | General population | Yes | 72/70 | 55/60 | 38 | 100000 IU every fifteen days vs. placebo: supplement duration is 3 months | Chemiluminescence | No |
| Grønborg et al. (2019)[42] | Danish | General population | No | 32/34 | 0/0 | 66 | 1200 IU daily vs. placebo: supplement duration is 3 months | LC-MS | Yes |
| Hajimohammadi et al. (2017)[43] | NA | Diseased population | No | 53/52 | 48/38 | 100 | 500 IU daily vs. placebo: supplement duration is 12 weeks | LC-MS | No |
| Hansen et al.  (2015)[44] | USA | General population | Yes | 60/60/61 | 0/0/0 | 221 | 800 IU/50000 IU every fifteen days vs. placebo: supplement duration is 60 days | LC-MS | No |
| Havens et al.  (2012)[45] | USA&Puerto Rico | Diseased population | No | 21/21 | 62/63 | 178 | 50000 IU monthly vs. placebo: supplement duration is 12 weeks | Radioimmunoassay | No |
| Himmelstein et al. (1990)[46] | USA | General population | No | 81/81 | 17/17 | 30 | 2000 IU daily vs. placebo: supplement duration is 7 weeks | others | No |
| Hin et al.  (2017)[47] | UK | General population | No | 71/72/72 | 51/51/50 | 305 | 2000 IU/4000 IU daily vs. placebo: supplement duration is 12 months | others | No |
| Holick et al.  (2008)[48] | USA | General population | No | 40/41 | 35/21 | 34 | 1000 IU daily vs. placebo: supplement duration is 11 weeks | LC-MS | No |
| Hurst et al.  (2010)[49] | New Zealand | Diseased population | Yes | 42/42 | 0/0 | 81 | 4000 IU daily vs. placebo: supplement duration is 6 months | Radioimmunoassay | No |
| Hussain et al.  (2019)[50] | Pakistan | Diseased population | No | 27/29 | 63/66 | 102 | 50000 IU weekly vs. placebo: supplement duration is 12 weeks | NA | No |
| Irandoust et al. (2017)[51] | Iran | Diseased population | Yes | 43/43 | 0/0 | 30 | 2000 IU daily vs. placebo: supplement duration is 12 weeks | others | Yes |
| Jafari et al.  (2016)[52] | Iran | Diseased population | No | 58/57 | 0/0 | 59 | 2000 IU daily vs. placebo: supplement duration is 12 weeks | ELISA | No |
| Jamilian et al.  (2017)[53] | Iran | Diseased population | No | 28/26/25 | 0/0/0 | 90 | 1000 IU/4000 IU daily vs. placebo: supplement duration is 12 weeks | ELISA | Yes |
| Javed et al.  (2019)[54] | UK | Diseased population | Yes | 29/29 | 0/0 | 37 | 3200 IU daily vs. placebo: supplement duration is 3 months | LC-MS | No |
| Jebreal et al.  (2020)[55] | Iran | General population | Yes | 70/67 | 51/25 | 81 | 50000 IU weekly vs. placebo: supplement duration is 12 weeks | Chemiluminescence | No |
| Kamelian et al. (2018)[56] | Iran | General population | Yes | 39/41 | 37/31 | 119 | 50000 IU weekly vs. placebo: supplement duration is 12 weeks | Chemiluminescence | No |
| Karefylakis et al. (2018)[57] | Sweden | Diseased population | No | 50/50 | 100/100 | 38 | 2000 IU daily vs. placebo: supplement duration is 6 months | LC-MS | No |
| Kaviani et al.  (2020)[58] | Iran | Diseased population | No | 43/43 | 18/4 | 56 | 50000 IU biweekly vs. placebo: supplement duration is 8 weeks | ELISA | No |
| Khan et al.  (2017)[59] | USA | Diseased population | No | 61/62 | 0/0 | 138 | 10000 IU 3 times a week vs. placebo: supplement duration is 24 weeks | LC-MS | Yes |
| Lerchbaum et al. (2017)[60] | Austria | General population | No | 34/38 | 100/100 | 98 | 20000 IU weekly vs. placebo: supplement duration is 4 weeks | LC-MS | No |
| Lerchbaum et al. (2019)[61] | Austria | General population | No | 48/50 | 100/100 | 94 | 20000 IU weekly vs. placebo: supplement duration is 12 weeks | LC-MS | No |
| Li-Ng et al.  (2009)[62] | USA | Diseased population | No | 59/58 | 19/22 | 148 | 2000 IU daily vs. placebo: supplement duration is 12 weeks | Radioimmunoassay | No |
| Longenecker et al. (2012)[63] | USA | Diseased population | Yes | 47/40 | 83/67 | 44 | 4000 IU daily vs. placebo: supplement duration is 12 weeks | ELISA | No |
| Lotfi‐Dizaji et al. (2019)[64] | Iran | Diseased population | Yes | 35/35 | 46/41 | 44 | 50000 IU weekly vs. placebo: supplement duration is 12 weeks | ELISA | No |
| Majid et al.  (2018)[65] | Iran | Diseased population | No | 38/36 | 25/22 | 89 | 50000 IU biweekly vs. placebo: supplement duration is 8 weeks | ELISA | No |
| Makariou et al. (2017)[66] | Greece | Diseased population | No | 52/51 | 44/60 | 50 | 2000 IU daily vs. placebo: supplement duration is 3 months | ELISA | No |
| Makariou et al. (2019)[67] | Greece | Diseased population | No | 52/53 | 60/40 | 50 | 2000 IU daily vs. placebo: supplement duration is 3 months | ELISA | No |
| Maktabi et al.  (2017)[68] | Iran | Diseased population | Yes | 22/23 | 0/0 | 70 | 50000 IU biweekly vs. placebo: supplement duration is 12 weeks | NA | No |
| Malhotra et al.  (2009)[69] | India | General population | No | 27/26 | 0/0 | 91 | 60000 IU monthly vs. placebo: supplement duration is 3 months | Radioimmunoassay | No |
| Mannheimer et al. (2015)[70] | Stockholm | Diseased population | No | 66/66 | 50/55 | 43 | 30000 IU weekly vs. placebo: supplement duration is 8 weeks | NA | No |
| Markland et al. (2016)[71] | USA | Diseased population | Yes | 61/61 | 0/0 | 45 | 50000 IU weekly vs. placebo: supplement duration is 12 weeks | NA | No |
| Markland et al. (2019)[72] | USA | General population | Yes | 61/60 | 0/0 | 49 | 50000 IU weekly vs. placebo: supplement duration is 12 weeks | NA | No |
| Martineau et al. (2015)[73] | UK | Diseased population | No | 49/46 | 44/43 | 250 | 120000 IU bimonthly vs. placebo: supplement duration is 12 months | LC-MS | No |
| Martineau et al. (2015)[74] | UK | Diseased population | No | 65/65 | 61/58 | 227 | 120000 IU bimonthly vs. placebo: supplement duration is 2 months | LC-MS | No |
| Mason et al.  (2016)[75] | USA | Diseased population | Yes | 60/60 | 0/0 | 187 | 2000 IU daily vs. placebo: supplement duration is 12 months | Chemiluminescence | No |
| Mazahery et al. (2015)[76] | New Zealand | General population | No | 36/35/37 | 0/0/0 | 61 | 50000 IU/100000 IU bimonthly vs. placebo: supplement duration is 6 months | Chemiluminescence | No |
| Mohammadi et al. (2016)[77] | Iran | Diseased population | No | 39/41 | 39/36 | 53 | 50000 IU weekly vs. placebo: supplement duration is 3 months | ELISA | No |
| Mony et al.  (2020)[78] | Indian | Diseased population | Yes | 39/37 | 22/20 | 120 | 60000 IU biweekly vs. placebo: supplement duration is 12 weeks | Chemiluminescence | No |
| Ng et al.  (2014)[79] | USA | General population | No | 51/50/51/51 | 33/34/35/27 | 292 | 1000 IU/2000 IU/4000 IU daily vs. placebo: supplement duration is 3 months | Radioimmunoassay | No |
| Niroomand et al. (2019)[80] | Iran | Diseased population | Yes | 45/48 | 25/22 | 118 | 50000 IU weekly vs. placebo: supplement duration is 3 months | Radioimmunoassay | No |
| Nodehi et al.  (2019)[81] | Iran | Diseased population | No | 36/36 | 0/0 | 34 | 50000 IU weekly vs. placebo: supplement duration is 12 weeks | ELISA | Yes |
| Omidian et al.  (2019)[82] | Tehran | Diseased population | No | 52/51 | 52/48 | 46 | 4000 IU daily vs. placebo: supplement duration is 12 weeks | NA | No |
| Patwardhan et al. (2017)[83] | India | General population | Yes | 48/48 | 0/0 | 76 | 1000 IU daily vs. placebo: supplement duration is 3 months | ELISA | No |
| Poel et al.  (2015)[84] | Netherland | Diseased population | No | 67/67 | 62/68 | 261 | 50000 IU monthly vs. placebo: supplement duration is 6 months | Radioimmunoassay | No |
| Qin et al.  (2015)[85] | China | Diseased population | No | 68/68 | 57/54 | 51 | 2000 IU daily vs. placebo: supplement duration is 3 months | ELISA | No |
| Rad et al.  (2014)[86] | Iran | Diseased population | No | 50/50 | 70/54 | 58 | 4000 IU daily vs. placebo: supplement duration is 2 months | Chemiluminescence | No |
| Ramezani Ahmadi et al. (2020)[87] | Iran | General population | No | 24/25 | 100/100 | 40 | 2000 IU daily vs. placebo: supplement duration is 12 weeks | LC-MS | No |
| Rashad et al.  (2020)[88] | Egypt | Diseased population | No | 26/26 | 0/0 | 95 | 42000 IU weekly vs. placebo: supplement duration is 12 weeks | ELISA | Yes |
| Raya et al.  (2013)[89] | Egypt | Diseased population | No | 39/39 | 15/15 | 267 | 2000 IU daily vs. placebo: supplement duration is 12 months | others | No |
| Razzaghi et al. (2017)[90] | NA | Diseased population | No | 59/60 | 73/73 | 60 | 50000 IU biweekly vs. placebo: supplement duration is 12 weeks | ELISA | No |
| Roosta et al.  (2018)[91] | Iran | Diseased population | No | 41/38 | 0/0 | 66 | 50000 IU every 25days vs. placebo: supplement duration is 3 months | ELISA | No |
| Sadiya et al.  (2014)[92] | United Arab Emirates | Diseased population | No | 49/48 | 20/17 | 87 | 6000 IU daily vs. placebo: supplement duration is 3 months | Chemiluminescence | No |
| Salehpour et al. (2012)[93] | Iran | Diseased population | No | 38/37 | 0/0 | 77 | 1000 IU daily vs. placebo: supplement duration is 12 weeks | others | No |
| Sfidvajani et al. (2017)[94] | Iran | Diseased population | Yes | 28/28 | 0/0 | 54 | 50000 IU weekly vs. placebo: supplement duration is 12 weeks | ELISA | Yes |
| Shalom et al.  (2008)[95] | Israel | Diseased population | No | 79/81/91 | 0/0/0 | 48 | 1500 IU daily / 10500 IU weekly / 45000 IU monthly: supplement duration is 8 weeks | Radioimmunoassay | No |
| Smith et al.  (2009)[96] | NA | General population | No | 43/44/42/39 | 68/43/72/83 | 62 | 400 IU/1000 IU/2000 IU daily vs. placebo: supplement duration is 13 weeks | ELISA | No |
| Sollid et al.  (2014)[97] | Norway | General population | No | 62/62 | 63/60 | 484 | 20000 IU weekly vs. placebo: supplement duration is 6 months | Chemiluminescence | No |
| Tabassi et al.  (2017)[98] | Iran | Diseased population | No | 42/43 | 0/0 | 60 | 50000 IU biweekly vs. placebo: supplement duration is 12 weeks | ELISA | No |
| Takács et al.  (2017)[99] | Hungary | General population | Yes | 51/56/52 | 14/22/18 | 61 | 1000 IU daily vs. 7000 IU weekly vs. 30000 IU monthly: supplement duration is 90 days | Chemiluminescence | No |
| Talaei et al.  (2018)[100] | Iran | Diseased population | No | 38/37 | NA/ | 201 | 50000 IU weekly vs. placebo: supplement duration is 12 weeks | ELISA | No |
| Tepper et al.  (2016)[101] | Israel | General population | Yes | NA | 100/100 | 130 | 100000 IU bimonthly vs. placebo: supplement duration is 12 months | Chemiluminescence | No |
| Thani et al.  (2019)[102] | Qatar | General population | No | 46/45 | 83/84 | 132 | 30000 IU weekly vs. placebo: supplement duration is 6 months | Chemiluminescence | No |
| Todd et al.  (2017)[103] | Northern Ireland | General population | No | 20/20 | 30/55 | 42 | 3000 IU daily vs. placebo: supplement duration is 12 weeks | LC-MS | No |
| Toss et al.  (2012)[104] | Sweden | General population | No | 70/70 | 29/29 | 45 | 1600 IU daily vs. placebo: supplement duration is 6 months | LC-MS | Yes |
| Tran et al.  (2012)[105] | Australia | General population | No | 60-84 | NA/ | 644 | 30000 IU/ 60000 IU monthly vs. placebo: supplement duration is 12 months | Chemiluminescence | No |
| Trummer et al. (2018)[106] | Austria. | Diseased population | No | 25/27 | 0/0 | 123 | 20000 IU weekly vs. placebo: supplement duration is 24 weeks | LC-MS | No |
| Trummer et al. (2020)[107] | Austria | General population | No | 36/36 | 0/0 | 126 | 20000 IU weekly vs. placebo: supplement duration is 24 weeks | LC-MS | No |
| Vahedpoor et al. (2017)[108] | Iran | General population | No | 37/39 | 0/0 | 58 | 50000 IU biweekly vs. placebo: supplement duration is 6 months | ELISA | No |
| Vahedpoor et al. (2018)[109] | Iran | Diseased population | No | 42/40 | 0/0 | 58 | 50000 IU biweekly vs. placebo: supplement duration is 6 months | ELISA | No |
| Wagner. et al  (2016)[110] | Sweden | General population | No | 68/67 | 57/50 | 43 | 30000 IU weekly ***vs.*** placebo; supplement duration is 8 weeks | Chemiluminescence | No |
| Wali et al.  (2019)[111] | Saudi Arabia | Diseased population | No | 43/42 | 65/72 | 22 | 50000 IU weekly vs. placebo: supplement duration is 12 weeks | NA | No |
| Wang et al.  (2020)[112] | China | Diseased population | No | 52/51 | 39/48 | 136 | 2000 IU daily vs. placebo: supplement duration is 2 months | others | No |
| Westerberg et al. (2018)[113] | Sweden | Diseased population | No | 63/64 | 80/56 | 89 | 8000 IU daily vs. placebo: supplement duration is 12 weeks | LC-MS | No |
| Yosaee et al.  (2020)[114] | Iran | Diseased population | No | 38/37 | 79/81 | 61 | 2000 IU daily vs. placebo: supplement duration is 12 weeks | ELISA | No |
| Zarrin et al.  (2017)[115] | Iran | General population | No | 48/48 | 49/47 | 104 | 1000 IU daily vs. placebo: supplement duration is 3 months | ELISA | No |
| Zhou et al.  (2015)[116] | China | Diseased population | No | NA | 52/71 | 56 | 1000 IU daily vs. placebo: supplement duration is 12 weeks | Radioimmunoassay | No |
| * Data separated by slashes represented data from the corresponding intervention and control groups in order. | | | | | | | | | |

1. Abdollahi R, Abiri B, Sarbakhsh P, Kashanian M, Vafa M. The Effect of Vitamin D Supplement Consumption on Premenstrual Syndrome in Vitamin D-Deficient Young Girls: A Randomized, Double-Blind, Placebo-Controlled Clinical Trial. Complement Med Res. 2019;26(5):336-42. Epub 2019/05/20. doi: 10.1159/000500016. PubMed PMID: 31104056.

2. Agarwal N, Mithal A, Dhingra V, Kaur P, Godbole MM, Shukla M. Effect of two different doses of oral cholecalciferol supplementation on serum 25-hydroxy-vitamin D levels in healthy Indian postmenopausal women: A randomized controlled trial. Indian journal of endocrinology and metabolism. 2013;17(5):883-9. Epub 2013/10/02. doi: 10.4103/2230-8210.117237. PubMed PMID: 24083171; PubMed Central PMCID: PMCPMC3784873.

3. Al-Bayyari N, Al-Domi H, Zayed F, Hailat R, Eaton A. Androgens and hirsutism score of overweight women with polycystic ovary syndrome improved after vitamin D treatment: A randomized placebo controlled clinical trial. Clin Nutr. 2021;40(3):870-8. Epub 2020/10/05. doi: 10.1016/j.clnu.2020.09.024. PubMed PMID: 33010974.

4. Aloia J, Dhaliwal R, Mikhail M, Shieh A, Stolberg A, Ragolia L, et al. Free 25(OH)D and Calcium Absorption, PTH, and Markers of Bone Turnover. J Clin Endocrinol Metab. 2015;100(11):4140-5. Epub 2015/08/28. doi: 10.1210/jc.2015-2548. PubMed PMID: 26312580; PubMed Central PMCID: PMCPMC4702446.

5. Amani R, Abbasnezhad A, Hajiani E, Cheraghian B, Abdoli Z, Choghakhori R. Vitamin D3 Induced Decrease in IL-17 and Malondialdehyde, and Increase in IL-10 and Total Antioxidant Capacity Levels in Patients with Irritable Bowel Syndrome. Iran J Immunol. 2018;15(3):186-96. Epub 2018/09/25. doi: 10.22034/IJI.2018.39388. PubMed PMID: 30246694.

6. Ameri P, Giusti A, Boschetti M, Bovio M, Teti C, Leoncini G, et al. Vitamin D increases circulating IGF1 in adults: potential implication for the treatment of GH deficiency. Eur J Endocrinol. 2013;169(6):767-72. Epub 2013/09/06. doi: 10.1530/EJE-13-0510. PubMed PMID: 24005315.

7. Amorim S, Teixeira VH, Corredeira R, Cunha M, Maia B, Margalho P, et al. Creatine or vitamin D supplementation in individuals with a spinal cord injury undergoing resistance training: A double-blinded, randomized pilot trial. J Spinal Cord Med. 2018;41(4):471-8. Epub 2017/09/14. doi: 10.1080/10790268.2017.1372058. PubMed PMID: 28901216; PubMed Central PMCID: PMCPMC6055973.

8. Anaraki PV, Aminorroaya A, Amini M, Feizi A, Iraj B, Tabatabaei A. Effects of Vitamin D deficiency treatment on metabolic markers in Hashimoto thyroiditis patients. J Res Med Sci. 2017;22:5. Epub 2017/04/13. doi: 10.4103/1735-1995.199090. PubMed PMID: 28400827; PubMed Central PMCID: PMCPMC5361437.

9. Anyanwu AC, Fasanmade OA, Odeniyi IA, Iwuala S, Coker HB, Ohwovoriole AE. Effect of Vitamin D supplementation on glycemic control in Type 2 diabetes subjects in Lagos, Nigeria. Indian journal of endocrinology and metabolism. 2016;20(2):189-94. Epub 2016/04/05. doi: 10.4103/2230-8210.176345. PubMed PMID: 27042414; PubMed Central PMCID: PMCPMC4792019.

10. Ardabili HR, Gargari BP, Farzadi L. Vitamin D supplementation has no effect on insulin resistance assessment in women with polycystic ovary syndrome and vitamin D deficiency. Nutr Res. 2012;32(3):195-201. Epub 2012/04/03. doi: 10.1016/j.nutres.2012.02.001. PubMed PMID: 22464806.

11. Arjeh S, Darsareh F, Asl ZA, Azizi Kutenaei M. Effect of oral consumption of vitamin D on uterine fibroids: A randomized clinical trial. Complement Ther Clin Pract. 2020;39:101159. Epub 2020/05/08. doi: 10.1016/j.ctcp.2020.101159. PubMed PMID: 32379687.

12. Mielgo-Ayuso J, Calleja-Gonzalez J, Urdampilleta A, Leon-Guereno P, Cordova A, Caballero-Garcia A, et al. Effects of Vitamin D Supplementation on Haematological Values and Muscle Recovery in Elite Male Traditional Rowers. Nutrients. 2018;10(12). Epub 2018/12/14. doi: 10.3390/nu10121968. PubMed PMID: 30545134; PubMed Central PMCID: PMCPMC6315465.

13. Barchetta I, Del Ben M, Angelico F, Di Martino M, Fraioli A, La Torre G, et al. No effects of oral vitamin D supplementation on non-alcoholic fatty liver disease in patients with type 2 diabetes: a randomized, double-blind, placebo-controlled trial. BMC Med. 2016;14:92. Epub 2016/06/30. doi: 10.1186/s12916-016-0638-y. PubMed PMID: 27353492; PubMed Central PMCID: PMCPMC4926287.

14. Barnes MS, Robson PJ, Bonham MP, Strain JJ, Wallace JM. Effect of vitamin D supplementation on vitamin D status and bone turnover markers in young adults. Eur J Clin Nutr. 2006;60(6):727-33. Epub 2006/01/05. doi: 10.1038/sj.ejcn.1602374. PubMed PMID: 16391584.

15. Bhagatwala J, Zhu H, Parikh SJ, Guo DH, Kotak I, Huang Y, et al. Dose and time responses of vitamin D biomarkers to monthly vitamin D3 supplementation in overweight/obese African Americans with suboptimal vitamin d status: a placebo controlled randomized clinical trial. BMC Obes. 2015;2:27. Epub 2015/07/29. doi: 10.1186/s40608-015-0056-2. PubMed PMID: 26217542; PubMed Central PMCID: PMCPMC4511449.

16. Shab-Bidar S, Neyestani TR, Djazayery A, Eshraghian MR, Houshiarrad A, Kalayi A, et al. Improvement of vitamin D status resulted in amelioration of biomarkers of systemic inflammation in the subjects with type 2 diabetes. Diabetes Metab Res Rev. 2012;28(5):424-30. Epub 2012/02/22. doi: 10.1002/dmrr.2290. PubMed PMID: 22344966.

17. Brisson J, Berube S, Diorio C, Masse B, Lemieux J, Duchesne T, et al. A Randomized Double-Blind Placebo-Controlled Trial of the Effect of Vitamin D3 Supplementation on Breast Density in Premenopausal Women. Cancer Epidemiol Biomarkers Prev. 2017;26(8):1233-41. Epub 2017/05/19. doi: 10.1158/1055-9965.EPI-17-0249. PubMed PMID: 28515107.

18. Vargas Buonfiglio LG, Cano M, Pezzulo AA, Vanegas Calderon OG, Zabner J, Gerke AK, et al. Effect of vitamin D3 on the antimicrobial activity of human airway surface liquid: preliminary results of a randomised placebo-controlled double-blind trial. BMJ Open Respir Res. 2017;4(1):e000211. Epub 2017/09/09. doi: 10.1136/bmjresp-2017-000211. PubMed PMID: 28883932; PubMed Central PMCID: PMCPMC5531307.

19. Carrillo AE, Flynn MG, Pinkston C, Markofski MM, Jiang Y, Donkin SS, et al. Vitamin D supplementation during exercise training does not alter inflammatory biomarkers in overweight and obese subjects. Eur J Appl Physiol. 2012;112(8):3045-52. Epub 2011/12/21. doi: 10.1007/s00421-011-2279-3. PubMed PMID: 22183086; PubMed Central PMCID: PMCPMC3417103.

20. He CS, Fraser WD, Tang J, Brown K, Renwick S, Rudland-Thomas J, et al. The effect of 14 weeks of vitamin D3 supplementation on antimicrobial peptides and proteins in athletes. Journal of sports sciences. 2016;34(1):67-74. Epub 2015/04/12. doi: 10.1080/02640414.2015.1033642. PubMed PMID: 25861808.

21. Chandler PD, Agboola F, Ng K, Scott JB, Drake BF, Bennett GG, et al. Reduction of Parathyroid Hormone with Vitamin D Supplementation in Blacks: A Randomized Controlled Trial. BMC Nutr. 2015;1. Epub 2016/02/10. doi: 10.1186/s40795-015-0024-8. PubMed PMID: 26858840; PubMed Central PMCID: PMCPMC4743037.

22. Chel V, Wijnhoven HA, Smit JH, Ooms M, Lips P. Efficacy of different doses and time intervals of oral vitamin D supplementation with or without calcium in elderly nursing home residents. Osteoporos Int. 2008;19(5):663-71. Epub 2007/09/18. doi: 10.1007/s00198-007-0465-2. PubMed PMID: 17874029; PubMed Central PMCID: PMCPMC2277446.

23. Cherniack EP, Florez HJ, Hollis BW, Roos BA, Troen BR, Levis S. The response of elderly veterans to daily vitamin D3 supplementation of 2,000 IU: a pilot efficacy study. J Am Geriatr Soc. 2011;59(2):286-90. Epub 2011/02/04. doi: 10.1111/j.1532-5415.2010.03242.x. PubMed PMID: 21288233.

24. Crew KD, Anderson GL, Hershman DL, Terry MB, Tehranifar P, Lew DL, et al. Randomized Double-Blind Placebo-Controlled Biomarker Modulation Study of Vitamin D Supplementation in Premenopausal Women at High Risk for Breast Cancer (SWOG S0812). Cancer Prev Res (Phila). 2019;12(7):481-90. Epub 2019/05/30. doi: 10.1158/1940-6207.CAPR-18-0444. PubMed PMID: 31138522; PubMed Central PMCID: PMCPMC6609474.

25. Dabbaghmanesh MH, Danafar F, Eshraghian A, Omrani GR. Vitamin D supplementation for the treatment of non-alcoholic fatty liver disease: A randomized double blind placebo controlled trial. Diabetes Metab Syndr. 2018;12(4):513-7. Epub 2018/03/29. doi: 10.1016/j.dsx.2018.03.006. PubMed PMID: 29588137.

26. Dadaei T, Safapoor MH, Asadzadeh Aghdaei H, Balaii H, Pourhoseingholi MA, Naderi N, et al. Effect of vitamin D3 supplementation on TNF-α serum level and disease activity index in Iranian IBD patients. Gastroenterology and hepatology from bed to bench. 2015;8(1):49-55. Epub 2015/01/15. PubMed PMID: 25584176; PubMed Central PMCID: PMCPMC4285932.

27. Damghanian.P, Javanbakht M, H, Honarvar N, M, Yousefirad E, Mohammadi H, Zarei M, et al. Effects of Vitamin D supplementation on 8-hydroxydeoxy guanosine and 3-nitrotyrosine in patients with type 2 diabetes: a randomized clinical trial. Nutrition. 2019;21(1):138-46. doi: 10.23751/pn.v21i1-S.5831.

28. Ebrahimkhani S, Ghavamzadeh S, Mehdizadeh A. The effects of vitamin D and curcuminoids supplementation on anthropometric measurements and blood pressure in type 2 diabetic patients with coexisting hypovitaminosis D: A double-blind, placebo-controlled randomized clinical trial. Clin Nutr ESPEN. 2020;37:178-86. Epub 2020/05/04. doi: 10.1016/j.clnesp.2020.02.017. PubMed PMID: 32359741.

29. El Hajj C, Chardigny JM, Boirie Y, Yammine K, Helou M, Walrand S. Effect of Vitamin D Treatment on Glucose Homeostasis and Metabolism in Lebanese Older Adults: A Randomized Controlled Trial. J Nutr Health Aging. 2018;22(9):1128-32. Epub 2018/11/01. doi: 10.1007/s12603-018-1083-8. PubMed PMID: 30379314.

30. El Hajj C, Walrand S, Helou M, Yammine K. Effect of Vitamin D Supplementation on Inflammatory Markers in Non-Obese Lebanese Patients with Type 2 Diabetes: A Randomized Controlled Trial. Nutrients. 2020;12(7). Epub 2020/07/15. doi: 10.3390/nu12072033. PubMed PMID: 32659891; PubMed Central PMCID: PMCPMC7400886.

31. Farag HAM, Hosseinzadeh-Attar MJ, Muhammad BA, Esmaillzadeh A, Bilbeisi AHE. Comparative effects of vitamin D and vitamin C supplementations with and without endurance physical activity on metabolic syndrome patients: a randomized controlled trial. Diabetol Metab Syndr. 2018;10:80. Epub 2018/11/21. doi: 10.1186/s13098-018-0384-8. PubMed PMID: 30455745; PubMed Central PMCID: PMCPMC6225665.

32. Farrokhian A, Raygan F, Bahmani F, Talari HR, Esfandiari R, Esmaillzadeh A, et al. Long-Term Vitamin D Supplementation Affects Metabolic Status in Vitamin D-Deficient Type 2 Diabetic Patients with Coronary Artery Disease. The Journal of nutrition. 2017;147(3):384-9. Epub 2017/01/27. doi: 10.3945/jn.116.242008. PubMed PMID: 28122931.

33. Foroozanfard F, Talebi M, Samimi M, Mehrabi S, Badehnoosh B, Jamilian M, et al. Effect of Two Different Doses of Vitamin D Supplementation on Metabolic Profiles of Insulin-Resistant Patients with Polycystic Ovary Syndrome: A Randomized, Double-Blind, Placebo-Controlled Trial. Horm Metab Res. 2017;49(8):612-7. Epub 2017/07/06. doi: 10.1055/s-0043-112346. PubMed PMID: 28679142.

34. Gao W, Tang H, Wang D, Zhou X, Song Y, Wang Z. Effect of short-term vitamin D supplementation after nonsurgical periodontal treatment: A randomized, double-masked, placebo-controlled clinical trial. J Periodontal Res. 2020;55(3):354-62. Epub 2020/01/22. doi: 10.1111/jre.12719. PubMed PMID: 31960448.

35. Geier A, Eichinger M, Stirnimann G, Semela D, Tay F, Seifert B, et al. Treatment of non-alcoholic steatohepatitis patients with vitamin D: a double-blinded, randomized, placebo-controlled pilot study. Scand J Gastroenterol. 2018;53(9):1114-20. Epub 2018/10/03. doi: 10.1080/00365521.2018.1501091. PubMed PMID: 30270688.

36. Ghaderi A, Banafshe HR, Motmaen M, Rasouli-Azad M, Bahmani F, Asemi Z. Clinical trial of the effects of vitamin D supplementation on psychological symptoms and metabolic profiles in maintenance methadone treatment patients. Prog Neuropsychopharmacol Biol Psychiatry. 2017;79(Pt B):84-9. Epub 2017/06/24. doi: 10.1016/j.pnpbp.2017.06.016. PubMed PMID: 28642082.

37. Ghaderi A, Rasouli-Azad M, Farhadi MH, Mirhosseini N, Motmaen M, Pishyareh E, et al. Exploring the Effects of Vitamin D Supplementation on Cognitive Functions and Mental Health Status in Subjects Under Methadone Maintenance Treatment. J Addict Med. 2020;14(1):18-25. Epub 2019/05/31. doi: 10.1097/ADM.0000000000000550. PubMed PMID: 31145174.

38. Ghaderi A, Banafshe H, Aghadavod E, Gholami M, Asemi Z, Mesdaghinia A. The Effects of Vitamin D Supplementation on Withdrawal Symptoms and the Expression of Inflammatory Cytokines and Insulin in Patients Under Methadone Maintenance Treatment: A Randomized, Double-Blind, Placebo-Controlled Trial. Iranian Journal of Psychiatry and Behavioral Sciences. 2020;14(1). doi: 10.5812/ijpbs.86969.

39. Ghorbani Z, Rafiee P, Fotouhi A, Haghighi S, Rasekh Magham R, Ahmadi ZS, et al. The effects of vitamin D supplementation on interictal serum levels of calcitonin gene-related peptide (CGRP) in episodic migraine patients: post hoc analysis of a randomized double-blind placebo-controlled trial. J Headache Pain. 2020;21(1):22. Epub 2020/02/26. doi: 10.1186/s10194-020-01090-w. PubMed PMID: 32093657; PubMed Central PMCID: PMCPMC7041277.

40. Glendenning P, Zhu K, Inderjeeth C, Howat P, Lewis JR, Prince RL. Effects of three-monthly oral 150,000 IU cholecalciferol supplementation on falls, mobility, and muscle strength in older postmenopausal women: a randomized controlled trial. J Bone Miner Res. 2012;27(1):170-6. Epub 2011/10/01. doi: 10.1002/jbmr.524. PubMed PMID: 21956713.

41. Goncalves-Mendes N, Talvas J, Duale C, Guttmann A, Corbin V, Marceau G, et al. Impact of Vitamin D Supplementation on Influenza Vaccine Response and Immune Functions in Deficient Elderly Persons: A Randomized Placebo-Controlled Trial. Front Immunol. 2019;10:65. Epub 2019/02/26. doi: 10.3389/fimmu.2019.00065. PubMed PMID: 30800121; PubMed Central PMCID: PMCPMC6375825.

42. Gronborg IM, Tetens I, Andersen EW, Kristensen M, Larsen REK, Tran TLL, et al. Effect of vitamin D fortified foods on bone markers and muscle strength in women of Pakistani and Danish origin living in Denmark: a randomised controlled trial. Nutr J. 2019;18(1):82. Epub 2019/12/04. doi: 10.1186/s12937-019-0504-9. PubMed PMID: 31791333; PubMed Central PMCID: PMCPMC6889210.

43. Hajimohammadi M, Shab-Bidar S, Neyestani TR. Consumption of vitamin D-fortified yogurt drink increased leptin and ghrelin levels but reduced leptin to ghrelin ratio in type 2 diabetes patients: a single blind randomized controlled trial. European journal of nutrition. 2017;56(6):2029-36. Epub 2017/02/24. doi: 10.1007/s00394-017-1397-z. PubMed PMID: 28229278.

44. Hansen KE, Johnson RE, Chambers KR, Johnson MG, Lemon CC, Vo TN, et al. Treatment of Vitamin D Insufficiency in Postmenopausal Women: A Randomized Clinical Trial. JAMA Intern Med. 2015;175(10):1612-21. Epub 2015/08/04. doi: 10.1001/jamainternmed.2015.3874. PubMed PMID: 26237520; PubMed Central PMCID: PMCPMC4594209.

45. Havens PL, Mulligan K, Hazra R, Flynn P, Rutledge B, Van Loan MD, et al. Serum 25-hydroxyvitamin D response to vitamin D3 supplementation 50,000 IU monthly in youth with HIV-1 infection. J Clin Endocrinol Metab. 2012;97(11):4004-13. Epub 2012/08/31. doi: 10.1210/jc.2012-2600. PubMed PMID: 22933542; PubMed Central PMCID: PMCPMC3485594.

46. Himmelstein S, Clemens TL, Rubin A, Lindsay R. Vitamin D supplementation in elderly nursing home residents increases 25(OH)D but not 1,25(OH)2D. Am J Clin Nutr. 1990;52(4):701-6. Epub 1990/10/01. doi: 10.1093/ajcn/52.4.701. PubMed PMID: 2403063.

47. Hin H, Tomson J, Newman C, Kurien R, Lay M, Cox J, et al. Optimum dose of vitamin D for disease prevention in older people: BEST-D trial of vitamin D in primary care. Osteoporos Int. 2017;28(3):841-51. Epub 2016/12/18. doi: 10.1007/s00198-016-3833-y. PubMed PMID: 27986983; PubMed Central PMCID: PMCPMC5306173.

48. Holick MF, Biancuzzo RM, Chen TC, Klein EK, Young A, Bibuld D, et al. Vitamin D2 is as effective as vitamin D3 in maintaining circulating concentrations of 25-hydroxyvitamin D. J Clin Endocrinol Metab. 2008;93(3):677-81. Epub 2007/12/20. doi: 10.1210/jc.2007-2308. PubMed PMID: 18089691; PubMed Central PMCID: PMCPMC2266966.

49. von Hurst PR, Stonehouse W, Coad J. Vitamin D supplementation reduces insulin resistance in South Asian women living in New Zealand who are insulin resistant and vitamin D deficient - a randomised, placebo-controlled trial. Br J Nutr. 2010;103(4):549-55. Epub 2009/09/29. doi: 10.1017/S0007114509992017. PubMed PMID: 19781131.

50. Hussain M, Iqbal J, Malik SA, Waheed A, Shabnum S, Akhtar L, et al. Effect of vitamin D supplementation on various parameters in non-alcoholic fatty liver disease patients. Pakistan journal of pharmaceutical sciences. 2019;32(3 Special):1343-8. Epub 2019/09/26. PubMed PMID: 31551213.

51. Irandoust K, Taheri M. The Effect of Vitamin D supplement and Indoor Vs Outdoor Physical Activity on Depression of Obese Depressed Women. Asian Journal of Sports Medicine. 2017;In Press(In Press). doi: 10.5812/asjsm.13311.

52. Jafari T, Faghihimani E, Feizi A, Iraj B, Javanmard SH, Esmaillzadeh A, et al. Effects of vitamin D-fortified low fat yogurt on glycemic status, anthropometric indexes, inflammation, and bone turnover in diabetic postmenopausal women: A randomised controlled clinical trial. Clin Nutr. 2016;35(1):67-76. Epub 2015/03/22. doi: 10.1016/j.clnu.2015.02.014. PubMed PMID: 25794439.

53. Jamilian M, Foroozanfard F, Rahmani E, Talebi M, Bahmani F, Asemi Z. Effect of Two Different Doses of Vitamin D Supplementation on Metabolic Profiles of Insulin-Resistant Patients with Polycystic Ovary Syndrome. Nutrients. 2017;9(12). Epub 2017/12/01. doi: 10.3390/nu9121280. PubMed PMID: 29186759; PubMed Central PMCID: PMCPMC5748731.

54. Javed Z, Papageorgiou M, Deshmukh H, Kilpatrick ES, Mann V, Corless L, et al. A Randomized, Controlled Trial of Vitamin D Supplementation on Cardiovascular Risk Factors, Hormones, and Liver Markers in Women with Polycystic Ovary Syndrome. Nutrients. 2019;11(1). Epub 2019/01/20. doi: 10.3390/nu11010188. PubMed PMID: 30658483; PubMed Central PMCID: PMCPMC6356309.

55. Jebreal Azimzadeh M, Shidfar F, Jazayeri S, Hosseini AF, Ranjbaran F. Effect of vitamin D supplementation on klotho protein, antioxidant status and nitric oxide in the elderly: A randomized, double-blinded, placebo-controlled clinical trial. European Journal of Integrative Medicine. 2020;35. doi: 10.1016/j.eujim.2020.101089.

56. Kamelian T, Saki F, Jeddi M, Dabbaghmanesh MH, Omrani GHR. Effect of Cholecalciferol therapy on serum FGF23 in vitamin D deficient patients: a randomized clinical trial. J Endocrinol Invest. 2018;41(3):299-306. Epub 2017/08/11. doi: 10.1007/s40618-017-0739-2. PubMed PMID: 28795342.

57. Karefylakis C, Sarnblad S, Ariander A, Ehlersson G, Rask E, Rask P. Effect of Vitamin D supplementation on body composition and cardiorespiratory fitness in overweight men-a randomized controlled trial. Endocrine. 2018;61(3):388-97. Epub 2018/07/07. doi: 10.1007/s12020-018-1665-6. PubMed PMID: 29978375; PubMed Central PMCID: PMCPMC6105237.

58. Kaviani M, Nikooyeh B, Zand H, Yaghmaei P, Neyestani TR. Effects of vitamin D supplementation on depression and some involved neurotransmitters. J Affect Disord. 2020;269:28-35. Epub 2020/03/29. doi: 10.1016/j.jad.2020.03.029. PubMed PMID: 32217340.

59. Khan QJ, Kimler BF, Reddy PS, Sharma P, Klemp JR, Nydegger JL, et al. Randomized trial of vitamin D3 to prevent worsening of musculoskeletal symptoms in women with breast cancer receiving adjuvant letrozole. The VITAL trial. Breast Cancer Res Treat. 2017;166(2):491-500. Epub 2017/08/05. doi: 10.1007/s10549-017-4429-8. PubMed PMID: 28770449.

60. Lerchbaum E, Pilz S, Trummer C, Schwetz V, Pachernegg O, Heijboer AC, et al. Vitamin D and Testosterone in Healthy Men: A Randomized Controlled Trial. The Journal of Clinical Endocrinology & Metabolism. 2017;102(11):4292-302. doi: 10.1210/jc.2017-01428.

61. Lerchbaum E, Trummer C, Theiler-Schwetz V, Kollmann M, Wolfler M, Heijboer AC, et al. Effects of vitamin D supplementation on androgens in men with low testosterone levels: a randomized controlled trial. European journal of nutrition. 2019;58(8):3135-46. Epub 2018/11/22. doi: 10.1007/s00394-018-1858-z. PubMed PMID: 30460609; PubMed Central PMCID: PMCPMC6842386.

62. Li-Ng M, Aloia JF, Pollack S, Cunha BA, Mikhail M, Yeh J, et al. A randomized controlled trial of vitamin D3 supplementation for the prevention of symptomatic upper respiratory tract infections. Epidemiol Infect. 2009;137(10):1396-404. Epub 2009/03/20. doi: 10.1017/S0950268809002404. PubMed PMID: 19296870.

63. Longenecker CT, Hileman CO, Carman TL, Ross AC, Seydafkan S, Brown TT, et al. Vitamin D supplementation and endothelial function in vitamin D deficient HIV-infected patients: a randomized placebo-controlled trial. Antivir Ther. 2012;17(4):613-21. Epub 2012/02/02. doi: 10.3851/IMP1983. PubMed PMID: 22293363; PubMed Central PMCID: PMCPMC3898848.

64. Lotfi-Dizaji L, Mahboob S, Aliashrafi S, Vaghef-Mehrabany E, Ebrahimi-Mameghani M, Morovati A. Effect of vitamin D supplementation along with weight loss diet on meta-inflammation and fat mass in obese subjects with vitamin D deficiency: A double-blind placebo-controlled randomized clinical trial. Clin Endocrinol (Oxf). 2019;90(1):94-101. Epub 2018/09/25. doi: 10.1111/cen.13861. PubMed PMID: 30246883.

65. Majid MS, Ahmad HS, Bizhan H, Hosein HZM, Mohammad A. The effect of vitamin D supplement on the score and quality of sleep in 20-50 year-old people with sleep disorders compared with control group. Nutr Neurosci. 2018;21(7):511-9. Epub 2017/05/06. doi: 10.1080/1028415X.2017.1317395. PubMed PMID: 28475473.

66. Makariou SE, Elisaf M, Challa A, Tentolouris N, Liberopoulos EN. No effect of vitamin D supplementation on cardiovascular risk factors in subjects with metabolic syndrome: a pilot randomised study. Archives of medical sciences Atherosclerotic diseases. 2017;2:e52-e60. Epub 2017/12/16. doi: 10.5114/amsad.2017.70504. PubMed PMID: 29242845; PubMed Central PMCID: PMCPMC5728078.

67. Makariou SE, Elisaf M, Challa A, Tellis CC, Tselepis AD, Liberopoulos EN. No effect of vitamin D administration plus dietary intervention on emerging cardiovascular risk factors in patients with metabolic syndrome. Journal of Nutrition & Intermediary Metabolism. 2019;16. doi: 10.1016/j.jnim.2019.100093.

68. Maktabi M, Chamani M, Asemi Z. The Effects of Vitamin D Supplementation on Metabolic Status of Patients with Polycystic Ovary Syndrome: A Randomized, Double-Blind, Placebo-Controlled Trial. Horm Metab Res. 2017;49(7):493-8. Epub 2017/07/06. doi: 10.1055/s-0043-107242. PubMed PMID: 28679140.

69. Malhotra N, Mithal A, Gupta S, Shukla M, Godbole M. Effect of vitamin D supplementation on bone health parameters of healthy young Indian women. Arch Osteoporos. 2009;4(1-2):47-53. Epub 2010/03/18. doi: 10.1007/s11657-009-0026-8. PubMed PMID: 20234858; PubMed Central PMCID: PMCPMC2836750.

70. Mannheimer B, Wagner H, Ostenson CG, Diczfalusy U. No impact of vitamin D on the CYP3A biomarker 4beta-hydroxycholesterol in patients with abnormal glucose regulation. PLoS One. 2015;10(4):e0121984. Epub 2015/04/04. doi: 10.1371/journal.pone.0121984. PubMed PMID: 25835492; PubMed Central PMCID: PMCPMC4383380.

71. Markland AD, Burgio K, Beasley T, Vaughan CP, Tangpricha V, Goode P. Vitamin D supplementation for urgency urinary incontinence in post-menopausal women: A pilot randomised clinical trial. Journal of Investigative Medicine. 2018;66(2):589. doi: 10.1136/jim-2017-000697.581.

72. Markland AD, Tangpricha V, Mark Beasley T, Vaughan CP, Richter HE, Burgio KL, et al. Comparing Vitamin D Supplementation Versus Placebo for Urgency Urinary Incontinence: A Pilot Study. J Am Geriatr Soc. 2019;67(3):570-5. Epub 2018/12/24. doi: 10.1111/jgs.15711. PubMed PMID: 30578542; PubMed Central PMCID: PMCPMC6403014.

73. Martineau AR, MacLaughlin BD, Hooper RL, Barnes NC, Jolliffe DA, Greiller CL, et al. Double-blind randomised placebo-controlled trial of bolus-dose vitamin D3 supplementation in adults with asthma (ViDiAs). Thorax. 2015;70(5):451-7. Epub 2015/03/01. doi: 10.1136/thoraxjnl-2014-206449. PubMed PMID: 25724847.

74. Martineau AR, James WY, Hooper RL, Barnes NC, Jolliffe DA, Greiller CL, et al. Vitamin D 3 supplementation in patients with chronic obstructive pulmonary disease (ViDiCO): a multicentre, double-blind, randomised controlled trial. The Lancet Respiratory Medicine. 2015;3(2):120-30. doi: 10.1016/s2213-2600(14)70255-3.

75. Mason C, Tapsoba JD, Duggan C, Imayama I, Wang CY, Korde L, et al. Effects of Vitamin D3 Supplementation on Lean Mass, Muscle Strength, and Bone Mineral Density During Weight Loss: A Double-Blind Randomized Controlled Trial. J Am Geriatr Soc. 2016;64(4):769-78. Epub 2016/04/10. doi: 10.1111/jgs.14049. PubMed PMID: 27060050; PubMed Central PMCID: PMCPMC4840082.

76. Mazahery H, Stonehouse W, von Hurst PR. The effect of monthly 50,000 IU or 100,000 IU vitamin D supplements on vitamin D status in premenopausal Middle Eastern women living in Auckland. Eur J Clin Nutr. 2015;69(3):367-72. Epub 2014/12/11. doi: 10.1038/ejcn.2014.264. PubMed PMID: 25491499.

77. Mohammadi SM, Eghbali SA, Soheilikhah S, Ashkezari SJ, Salami M, Afkhami-Ardekani M, et al. The effects of vitamin D supplementation on adiponectin level and insulin resistance in first-degree relatives of subjects with type 2 diabetes: a randomized double-blinded controlled trial. Electron Physician. 2016;8(9):2849-54. Epub 2016/10/30. doi: 10.19082/2849. PubMed PMID: 27790335; PubMed Central PMCID: PMCPMC5074741.

78. Mony A, Chandrashekar L, Rajappa M, Munisamy M, Sahoo JP, Selvarajan S. Effect of vitamin D supplementation on clinical outcome and biochemical profile in South Indian population with vitamin D-deficient chronic urticarial - A randomized double-blind placebo controlled trial. Clin Chim Acta. 2020;504:1-6. Epub 2020/01/12. doi: 10.1016/j.cca.2020.01.003. PubMed PMID: 31926152.

79. Ng K, Scott JB, Drake BF, Chan AT, Hollis BW, Chandler PD, et al. Dose response to vitamin D supplementation in African Americans: results of a 4-arm, randomized, placebo-controlled trial. Am J Clin Nutr. 2014;99(3):587-98. Epub 2013/12/26. doi: 10.3945/ajcn.113.067777. PubMed PMID: 24368437; PubMed Central PMCID: PMCPMC3927692.

80. Niroomand M, Fotouhi A, Irannejad N, Hosseinpanah F. Does high-dose vitamin D supplementation impact insulin resistance and risk of development of diabetes in patients with pre-diabetes? A double-blind randomized clinical trial. Diabetes Res Clin Pract. 2019;148:1-9. Epub 2018/12/26. doi: 10.1016/j.diabres.2018.12.008. PubMed PMID: 30583032.

81. Nodehi M, Ajami A, Izad M, Asgarian Omran H, Chahardoli R, Amouzegar A, et al. Effects of vitamin D supplements on frequency of CD4(+) T-cell subsets in women with Hashimoto's thyroiditis: a double-blind placebo-controlled study. Eur J Clin Nutr. 2019;73(9):1236-43. Epub 2019/01/31. doi: 10.1038/s41430-019-0395-z. PubMed PMID: 30696977.

82. Omidian M, Djalali M, Javanbakht MH, Eshraghian MR, Abshirini M, Omidian P, et al. Effects of vitamin D supplementation on advanced glycation end products signaling pathway in T2DM patients: a randomized, placebo-controlled, double blind clinical trial. Diabetol Metab Syndr. 2019;11:86. Epub 2019/11/02. doi: 10.1186/s13098-019-0479-x. PubMed PMID: 31673295; PubMed Central PMCID: PMCPMC6814978.

83. Patwardhan VG, Mughal ZM, Padidela R, Chiplonkar SA, Khadilkar VV, Khadilkar AV. Randomized Control Trial Assessing Impact of Increased Sunlight Exposure versus Vitamin D Supplementation on Lipid Profile in Indian Vitamin D Deficient Men. Indian journal of endocrinology and metabolism. 2017;21(3):393-8. Epub 2017/05/30. doi: 10.4103/ijem.IJEM_9_17. PubMed PMID: 28553593; PubMed Central PMCID: PMCPMC5434721.

84. Krul-Poel YH, Westra S, ten Boekel E, ter Wee MM, van Schoor NM, van Wijland H, et al. Effect of Vitamin D Supplementation on Glycemic Control in Patients With Type 2 Diabetes (SUNNY Trial): A Randomized Placebo-Controlled Trial. Diabetes Care. 2015;38(8):1420-6. Epub 2015/05/15. doi: 10.2337/dc15-0323. PubMed PMID: 25972575.

85. Qin XF, Zhao LS, Chen WR, Yin DW, Wang H. Effects of vitamin D on plasma lipid profiles in statin-treated patients with hypercholesterolemia: A randomized placebo-controlled trial. Clin Nutr. 2015;34(2):201-6. Epub 2014/05/23. doi: 10.1016/j.clnu.2014.04.017. PubMed PMID: 24844869.

86. Yousefi Rad E, Djalali M, Koohdani F, Saboor-Yaraghi AA, Eshraghian MR, Javanbakht MH, et al. The Effects of Vitamin D Supplementation on Glucose Control and Insulin Resistance in Patients with Diabetes Type 2: A Randomized Clinical Trial Study. Iranian journal of public health. 2014;43(12):1651-6. Epub 2015/07/15. PubMed PMID: 26171357; PubMed Central PMCID: PMCPMC4499086.

87. Ramezani Ahmadi A, Mohammadshahi M, Alizadeh A, Ahmadi Angali K, Jahanshahi A. Effects of vitamin D3 supplementation for 12 weeks on serum levels of anabolic hormones, anaerobic power, and aerobic performance in active male subjects: A randomized, double-blind, placebo-controlled trial. Eur J Sport Sci. 2020;20(10):1355-67. Epub 2020/01/21. doi: 10.1080/17461391.2020.1713218. PubMed PMID: 31957555.

88. Rashad NM, Abd El-Fatah AH, Lashin ME-B, Abomandour HG, Allam RM. Impact of vitamin D supplementation on cardio-metabolic status and androgen profile in women with polycystic ovary syndrome: placebo-controlled clinical trial. Middle East Fertility Society Journal. 2019;24(1). doi: 10.1186/s43043-019-0005-y.

89. Abou-Raya A, Abou-Raya S, Helmii M. The effect of vitamin D supplementation on inflammatory and hemostatic markers and disease activity in patients with systemic lupus erythematosus: a randomized placebo-controlled trial. J Rheumatol. 2013;40(3):265-72. Epub 2012/12/04. doi: 10.3899/jrheum.111594. PubMed PMID: 23204220.

90. Razzaghi R, Pourbagheri H, Momen-Heravi M, Bahmani F, Shadi J, Soleimani Z, et al. The effects of vitamin D supplementation on wound healing and metabolic status in patients with diabetic foot ulcer: A randomized, double-blind, placebo-controlled trial. J Diabetes Complications. 2017;31(4):766-72. Epub 2016/07/02. doi: 10.1016/j.jdiacomp.2016.06.017. PubMed PMID: 27363929.

91. Roosta S, Kharadmand M, Teymoori F, Birjandi M, Adine A, Falahi E. Effect of vitamin D supplementation on anthropometric indices among overweight and obese women: A double blind randomized controlled clinical trial. Diabetes Metab Syndr. 2018;12(4):537-41. Epub 2018/04/05. doi: 10.1016/j.dsx.2018.03.022. PubMed PMID: 29615318.

92. Sadiya A, Ahmed SM, Carlsson M, Tesfa Y, George M, Ali SH, et al. Vitamin D supplementation in obese type 2 diabetes subjects in Ajman, UAE: a randomized controlled double-blinded clinical trial. Eur J Clin Nutr. 2015;69(6):707-11. Epub 2014/11/20. doi: 10.1038/ejcn.2014.251. PubMed PMID: 25406966; PubMed Central PMCID: PMCPMC4458894.

93. Salehpour A, Shidfar F, Hosseinpanah F, Vafa M, Razaghi M, Hoshiarrad A, et al. Vitamin D3 and the risk of CVD in overweight and obese women: a randomised controlled trial. Br J Nutr. 2012;108(10):1866-73. Epub 2012/02/10. doi: 10.1017/S0007114512000098. PubMed PMID: 22317756.

94. Jafari-Sfidvajani S, Ahangari R, Hozoori M, Mozaffari-Khosravi H, Fallahzadeh H, Nadjarzadeh A. The effect of vitamin D supplementation in combination with low-calorie diet on anthropometric indices and androgen hormones in women with polycystic ovary syndrome: a double-blind, randomized, placebo-controlled trial. J Endocrinol Invest. 2018;41(5):597-607. Epub 2017/11/08. doi: 10.1007/s40618-017-0785-9. PubMed PMID: 29110281.

95. Ish-Shalom S, Segal E, Salganik T, Raz B, Bromberg IL, Vieth R. Comparison of daily, weekly, and monthly vitamin D3 in ethanol dosing protocols for two months in elderly hip fracture patients. J Clin Endocrinol Metab. 2008;93(9):3430-5. Epub 2008/06/12. doi: 10.1210/jc.2008-0241. PubMed PMID: 18544622.

96. Smith SM, Gardner KK, Locke J, Zwart SR. Vitamin D supplementation during Antarctic winter. Am J Clin Nutr. 2009;89(4):1092-8. Epub 2009/02/20. doi: 10.3945/ajcn.2008.27189. PubMed PMID: 19225122.

97. Sollid ST, Hutchinson MY, Fuskevag OM, Figenschau Y, Joakimsen RM, Schirmer H, et al. No effect of high-dose vitamin D supplementation on glycemic status or cardiovascular risk factors in subjects with prediabetes. Diabetes Care. 2014;37(8):2123-31. Epub 2014/06/21. doi: 10.2337/dc14-0218. PubMed PMID: 24947792.

98. Tabassi Z, Bagheri S, Samimi M, Gilasi HR, Bahmani F, Chamani M, et al. Clinical and Metabolic Response to Vitamin D Supplementation in Endometrial Hyperplasia: a Randomized, Double-Blind, Placebo-Controlled Trial. Horm Cancer. 2017;8(3):185-95. Epub 2017/03/12. doi: 10.1007/s12672-017-0290-9. PubMed PMID: 28283863.

99. Takacs I, Toth BE, Szekeres L, Szabo B, Bakos B, Lakatos P. Randomized clinical trial to comparing efficacy of daily, weekly and monthly administration of vitamin D3. Endocrine. 2017;55(1):60-5. Epub 2016/10/09. doi: 10.1007/s12020-016-1137-9. PubMed PMID: 27718150.

100. Talaei A, Ghorbani F, Asemi Z. The Effects of Vitamin D Supplementation on Thyroid Function in Hypothyroid Patients: A Randomized, Double-blind, Placebo-controlled Trial. Indian journal of endocrinology and metabolism. 2018;22(5):584-8. Epub 2018/10/09. doi: 10.4103/ijem.IJEM_603_17. PubMed PMID: 30294564; PubMed Central PMCID: PMCPMC6166548.

101. Tepper S, Shahar DR, Geva D, Ish-Shalom S. Differences in homeostatic model assessment (HOMA) values and insulin levels after vitamin D supplementation in healthy men: a double-blind randomized controlled trial. Diabetes Obes Metab. 2016;18(6):633-7. Epub 2016/02/20. doi: 10.1111/dom.12650. PubMed PMID: 26890031.

102. Al Thani M, Sadoun E, Sofroniou A, Jayyousi A, Baagar KAM, Al Hammaq A, et al. The effect of vitamin D supplementation on the glycemic control of pre-diabetic Qatari patients in a randomized control trial. BMC Nutr. 2019;5:46. Epub 2020/03/11. doi: 10.1186/s40795-019-0311-x. PubMed PMID: 32153959; PubMed Central PMCID: PMCPMC7050821.

103. Todd JJ, McSorley EM, Pourshahidi LK, Madigan SM, Laird E, Healy M, et al. Vitamin D3 supplementation using an oral spray solution resolves deficiency but has no effect on VO2 max in Gaelic footballers: results from a randomised, double-blind, placebo-controlled trial. European journal of nutrition. 2017;56(4):1577-87. Epub 2016/03/27. doi: 10.1007/s00394-016-1202-4. PubMed PMID: 27015912; PubMed Central PMCID: PMCPMC5486642.

104. Toss G, Magnusson P. Is a daily supplementation with 40 microgram vitamin D3 sufficient? A randomised controlled trial. European journal of nutrition. 2011;51(8):939-45. doi: 10.1007/s00394-011-0271-7.

105. Tran B, Armstrong BK, Carlin JB, Ebeling PR, English DR, Kimlin MG, et al. Recruitment and results of a pilot trial of vitamin D supplementation in the general population of Australia. J Clin Endocrinol Metab. 2012;97(12):4473-80. Epub 2012/10/16. doi: 10.1210/jc.2012-2682. PubMed PMID: 23066119.

106. Trummer C, Schwetz V, Kollmann M, Wolfler M, Munzker J, Pieber TR, et al. Effects of vitamin D supplementation on metabolic and endocrine parameters in PCOS: a randomized-controlled trial. European journal of nutrition. 2019;58(5):2019-28. Epub 2018/06/28. doi: 10.1007/s00394-018-1760-8. PubMed PMID: 29946756; PubMed Central PMCID: PMCPMC6647224.

107. Trummer C, Theiler-Schwetz V, Kollmann M, Wolfler M, Munzker J, Pilz S, et al. Effects of vitamin D supplementation on metabolic and endocrine parameters in healthy premenopausal women: A randomized controlled trial. Clin Nutr. 2020;39(3):718-26. Epub 2019/04/04. doi: 10.1016/j.clnu.2019.03.007. PubMed PMID: 30940404.

108. Vahedpoor Z, Jamilian M, Bahmani F, Aghadavod E, Karamali M, Kashanian M, et al. Effects of Long-Term Vitamin D Supplementation on Regression and Metabolic Status of Cervical Intraepithelial Neoplasia: a Randomized, Double-Blind, Placebo-Controlled Trial. Horm Cancer. 2017;8(1):58-67. Epub 2017/01/05. doi: 10.1007/s12672-016-0278-x. PubMed PMID: 28050798.

109. Vahedpoor Z, Mahmoodi S, Samimi M, Gilasi HR, Bahmani F, Soltani A, et al. Long-Term Vitamin D Supplementation and the Effects on Recurrence and Metabolic Status of Cervical Intraepithelial Neoplasia Grade 2 or 3: A Randomized, Double-Blind, Placebo-Controlled Trial. Ann Nutr Metab. 2018;72(2):151-60. Epub 2018/02/22. doi: 10.1159/000487270. PubMed PMID: 29466786.

110. Wagner H, Alvarsson M, Mannheimer B, Degerblad M, Östenson CG. No Effect of High-Dose Vitamin D Treatment on β-Cell Function, Insulin Sensitivity, or Glucose Homeostasis in Subjects With Abnormal Glucose Tolerance: A Randomized Clinical Trial. Diabetes Care. 2016;39(3):345-52. Epub 2016/01/21. doi: 10.2337/dc15-1057. PubMed PMID: 26786573.

111. Wali SO, Abaalkhail B, Alhejaili F, Pandi-Perumal SR. Efficacy of vitamin D replacement therapy in restless legs syndrome: a randomized control trial. Sleep Breath. 2019;23(2):595-601. Epub 2018/11/16. doi: 10.1007/s11325-018-1751-2. PubMed PMID: 30430372.

112. Wang CC, Tzeng IS, Su WC, Li CH, Lin HH, Yang CC, et al. The association of vitamin D with hepatitis B virus replication: Bystander rather than offender. J Formos Med Assoc. 2020;119(11):1634-41. Epub 2020/01/15. doi: 10.1016/j.jfma.2019.12.004. PubMed PMID: 31932201.

113. Westerberg PA, Sterner G, Ljunggren O, Isaksson E, Elvarson F, Dezfoolian H, et al. High doses of cholecalciferol alleviate the progression of hyperparathyroidism in patients with CKD Stages 3-4: results of a 12-week double-blind, randomized, controlled study. Nephrol Dial Transplant. 2018;33(3):466-71. Epub 2017/11/21. doi: 10.1093/ndt/gfx059. PubMed PMID: 29156056; PubMed Central PMCID: PMCPMC6018863.

114. Yosaee S, Soltani S, Esteghamati A, Motevalian SA, Tehrani-Doost M, Clark CCT, et al. Effects of zinc, vitamin D, and their co-supplementation on mood, serum cortisol, and brain-derived neurotrophic factor in patients with obesity and mild to moderate depressive symptoms: A phase II, 12-wk, 2x2 factorial design, double-blind, randomized, placebo-controlled trial. Nutrition. 2020;71:110601. Epub 2019/12/15. doi: 10.1016/j.nut.2019.110601. PubMed PMID: 31837640.

115. Zarrin R, Ayremlou P, Ghassemi F. The Effect of Vitamin D Supplementation on the Glycemic Status and the Percentage of Body Fat Mass in Adults with Prediabetes: A Randomized Clinical Trial. Iranian Red Crescent Medical Journal. 2016;19(3). doi: 10.5812/ircmj.41718.

116. Zhou W, Ye SD. Relationship between serum 25-hydroxyvitamin D and lower extremity arterial disease in type 2 diabetes mellitus patients and the analysis of the intervention of vitamin D. Journal of diabetes research. 2015;2015:815949. Epub 2015/04/30. doi: 10.1155/2015/815949. PubMed PMID: 25922846; PubMed Central PMCID: PMCPMC4397474.
