# Supplementary material for: Efficacy of intermittent versus daily vitamin D supplementation on improving circulating 25(OH)D concentration: a Bayesian network meta-analysis of randomized controlled trials
Source: Front Nutr. 2023 Aug 24;10:1168115. doi: 10.3389/fnut.2023.1168115 (PMC10488712; doi:10.3389/fnut.2023.1168115)
Supplement: Supplementary file 9 [file Table_9.DOCX]

| Table S4. Risk of bias assessment of included studies using RoB2 tool. | | | | | | | |
| --- | --- | --- | --- | --- | --- | --- | --- |
| Study | Random sequence generation, selection bias | Allocation concealment, Selection bias | Blinding of participants and personnel, performance bias | Blinding of outcome, assessment detection bias. | Incomplete outcome, data attrition bias | Selective reporting, reporting bias | Other bias |
| Abdollahi et al. (2019) | Low | Low | Low | Low | Low | Unclear | Low |
| Agarwal et al. (2013) | Low | Unclear | High | Unclear | Low | Unclear | High |
| Al-Bayyari et al. (2020) | Low | Low | Low | Unclear | Low | Low | Low |
| Aloia et al. (2015) | Low | Low | Low | Low | Low | Unclear | High |
| Amani et al. (2018) | Low | Low | Low | Unclear | Low | Low | Low |
| Ameri et al. (2013) | Unclear | High | High | High | Unclear | Unclear | High |
| Amorim et al. (2017) | Low | Low | Low | Low | Low | Low | High |
| Anaraki et al. (2017) | Low | Low | Low | Unclear | Low | Low | High |
| Anyanwu et al. (2016) | Unclear | Unclear | Unclear | Unclear | Low | Unclear | Low |
| Ardabili et al. (2012) | Low | Low | Low | Low | Low | Low | Low |
| Arjeh, S. et al. (2020) | Low | Low | Low | Low | Low | Low | High |
| Ayuso et al. (2018) | Low | Unclear | Low | Unclear | Low | Unclear | Unclear |
| Barchetta et al. (2016) | Low | Low | Low | Low | Low | Low | Low |
| Barnes et al. (2006) | Low | Unclear | Low | Low | Low | Unclear | High |
| Bhagatwala et al. (2015) | Low | Low | Low | Low | Low | Low | Low |
| Bidar et al. (2012) | Unclear | Unclear | Low | Unclear | Low | Low | Low |
| Brisson et al. (2017) | Low | Low | Low | Low | Low | Low | Low |
| Buonfiglio et al. (2017) | Low | Low | Low | Low | Low | Unclear | Unclear |
| Carrillo et al. (2012) | Unclear | Low | Low | Unclear | Unclear | Unclear | High |
| Cavalcante. H. et al (2015) | Low | Low | Unclear | Unclear | Unclear | Unclear | High |
| Chandler et al. (2015) | Low | Low | Low | Low | Low | Low | Low |
| Chel et al. (2008) | Low | High | High | Unclear | Low | Low | Low |
| Cherniack et al. (2011) | Low | Low | Low | Unclear | Low | Unclear | Low |
| Crew et al. (2019) | Low | Low | Low | Low | High | Low | Low |
| Dabbaghmanesh et al. (2018) | Low | Low | Low | Unclear | Low | Unclear | Unclear |
| Dadaei et al. (2015) | Low | Low | Low | Unclear | Low | Low | High |
| Damghanian et al. (2019) | Low | Low | Low | Unclear | Low | Low | Low |
| Ebrahimkhani et al. (2020) | Low | Low | Low | Low | Low | Low | Low |
| El Hajj et al. (2018) | Low | Low | Low | Low | Low | Low | Low |
| El Hajj et al. (2020) | Low | Low | Low | Low | Low | Low | Low |
| Farag et al. (2018) | Low | Low | High | Unclear | Low | Unclear | Low |
| Farrokhian et al. (2017) | Low | Low | Low | Low | Low | Low | Low |
| Foroozanfard et al. (2017) | Low | Low | Low | Unclear | Low | High | High |
| Gao et al. (2020) | Low | Low | Low | Unclear | Low | Low | High |
| Geier et al. (2018) | Unclear | Unclear | Low | Unclear | Low | Low | High |
| Ghaderi et al. (2017) | Low | Low | Low | Low | Low | Low | Low |
| Ghaderi et al. (2020) | Low | Low | Low | Unclear | Low | High | Unclear |
| Ghaderi et al. (2020) | Low | Low | Low | Unclear | Low | Low | Unclear |
| Ghorbani et al. (2020) | Low | Low | Low | Low | Low | Unclear | Low |
| Glendenning et al. (2012) | Low | Low | Low | Low | Low | Low | Low |
| Goncalves-Mendes et al. (2019) | Low | Low | Low | Low | Low | Low | High |
| Grønborg et al. (2019) | Low | Low | Low | Low | Low | Low | Low |
| Hajimohammadi et al. (2017) | Low | Low | Low | Low | Low | Low | Low |
| Hansen et al. (2015) | Low | Low | Low | Low | Low | Low | Low |
| Havens et al. (2012) | Unclear | Unclear | Low | Low | Low | Unclear | Unclear |
| Himmelstein et al. (1990) | Unclear | Unclear | Low | Unclear | Unclear | Unclear | High |
| Hin et al. (2017) | Low | Low | Low | Low | Low | Low | Low |
| Holick et al. (2008) | Unclear | Unclear | Low | Unclear | Unclear | Unclear | High |
| Hurst et al. (2010) | Low | Unclear | Low | High | Unclear | Low | Low |
| Hussain et al. (2019) | Low | Low | Low | Unclear | Low | Unclear | Low |
| Irandoust et al. (2017) | Unclear | High | High | High | Low | Unclear | High |
| Jafari et al. (2016) | Low | Low | Low | Low | Low | Low | Unclear |
| Jamilian et al. (2017) | Low | Low | Low | Unclear | Low | Low | Low |
| Javed et al. (2019) | Low | Unclear | Low | Unclear | Low | Low | High |
| Jebreal et al. (2020) | Low | Low | Low | Unclear | Low | Unclear | Low |
| Kamelian et al. (2018) | Low | Low | Low | Low | Low | Low | Low |
| Karefylakis et al. (2018) | Low | Low | Low | Unclear | Low | Unclear | Low |
| Kaviani et al. (2020) | Low | Low | Low | Low | Low | Low | Low |
| Khan et al. (2017) | Unclear | Unclear | Low | High | Low | Low | Unclear |
| Lerchbaum et al. (2017) | Low | Low | Low | Unclear | Low | Low | Unclear |
| Lerchbaum et al. (2019) | Low | Low | Low | Low | Low | Low | Low |
| Li-Ng et al. (2009) | Low | Low | Low | Low | Low | Unclear | Low |
| Longenecker et al. (2012) | Low | Low | Low | Low | Unclear | Unclear | High |
| Lotfi‐Dizaji et al. (2019) | Low | Low | Low | Unclear | Low | Low | High |
| Majid et al. (2018) | Low | Low | Low | Low | Low | Unclear | Low |
| Makariou et al. (2017) | Low | High | High | High | Unclear | Low | Unclear |
| Makariou et al. (2019) | Low | High | High | High | High | Low | Low |
| Maktabi et al. (2017) | Low | Low | Low | Unclear | Low | Unclear | Low |
| Malhotra et al. (2009) | Low | Unclear | Unclear | Unclear | Low | Unclear | High |
| Mannheimer et al. (2015) | Low | Low | Low | Unclear | Low | Low | High |
| Markland et al. (2016) | Low | Low | Low | Low | Low | Low | High |
| Markland et al. (2019) | Low | Low | Low | Unclear | Low | Low | High |
| Martineau et al. (2015) | Low | Low | Low | Low | Low | Low | Low |
| Martineau et al. (2015) | Low | Low | Low | Low | Low | Low | Low |
| Mason et al. (2016) | Low | Low | Low | Low | Low | Low | Low |
| Mazahery et al. (2015) | Low | Low | Low | Low | Low | Unclear | Low |
| Mohammadi et al. (2016) | Low | Low | Low | Unclear | Low | Low | Low |
| Mony et al. (2020) | Low | Low | Low | Unclear | Low | Low | Low |
| Ng et al. (2014) | Low | Low | Low | Low | Low | Low | Low |
| Niroomand et al. (2019) | Low | Low | Low | Low | Low | Low | High |
| Nodehi et al. (2019) | Low | Unclear | Low | Unclear | Low | Low | High |
| Omidian et al. (2019) | Low | Low | Low | Low | Low | Low | High |
| Patwardhan et al. (2017) | Low | High | High | High | Low | Unclear | Unclear |
| Poel et al. (2015) | Low | Low | Low | Low | Low | Low | Low |
| Qin et al. (2015) | Low | Low | Low | Low | Low | Low | Unclear |
| Rad et al. (2014) | Unclear | Unclear | Unclear | Unclear | Low | Low | Unclear |
| Ramezani Ahmadi et al. (2020) | Low | Low | Low | Unclear | Low | Low | Low |
| Rashad et al. (2020) | Low | High | High | Unclear | Low | Low | Low |
| Raya et al. (2013) | Low | Low | Low | Low | Low | Unclear | Low |
| Razzaghi et al. (2017) | Low | Low | Low | Low | Low | Low | Unclear |
| Roosta et al. (2018) | Low | Unclear | Low | Low | Low | Low | Unclear |
| Sadiya et al. (2014) | Low | Low | Low | Low | Low | Low | Low |
| Salehpour et al. (2012) | Unclear | Unclear | Low | Unclear | Low | Low | Unclear |
| Sfidvajani et al. (2017) | Low | Low | Low | Unclear | Low | Low | Low |
| Shalom et al. (2008) | Low | Low | High | Unclear | Unclear | Unclear | High |
| Smith et al. (2009) | Unclear | Low | Low | Unclear | Unclear | Unclear | High |
| Sollid et al. (2014) | Low | Low | Low | Unclear | Low | Low | Low |
| Tabassi et al. (2017) | Low | Low | Low | Low | Low | Low | High |
| Takács et al. (2017) | Low | High | High | High | Low | Low | Low |
| Talaei et al. (2018) | Low | Low | Low | Low | Low | Low | Low |
| Tepper et al. (2016) | Unclear | Low | Low | Low | Low | Low | Low |
| Thani et al. (2019) | Low | Low | Low | Unclear | High | Low | Low |
| Todd et al. (2017) | Unclear | Low | Low | Unclear | Low | Low | High |
| Toss et al. (2012) | Low | Low | Low | Low | Low | Low | Low |
| Tran et al. (2012) | Low | Low | Low | Unclear | Low | Low | Low |
| Trummer et al. (2018) | Low | Low | Low | Low | High | High | Low |
| Trummer et al. (2020) | Low | Low | Low | Unclear | Low | Low | Low |
| Vahedpoor et al. (2017) | Low | Low | Low | Low | Low | Low | Low |
| Vahedpoor et al. (2018) | Low | Low | Low | Low | Low | Low | Unclear |
| Wagner. H. et al (2016) | Low | Low | Low | Low | Low | Low | Low |
| Wali et al. (2019) | Low | Low | Low | Low | Unclear | Low | Low |
| Wang et al. (2020) | Low | High | High | High | Low | Unclear | Low |
| Westerberg et al. (2018) | Low | Low | Low | Low | Low | Low | Low |
| Yosaee et al. (2020) | Low | Low | Low | Low | Low | Low | Low |
| Zarrin et al. (2017) | Low | Unclear | Unclear | Unclear | Low | Low | Unclear |
| Zhou et al. (2015) | Unclear | Unclear | Unclear | Unclear | Unclear | Unclear | Unclear |
