# Supplementary material for: Efficacy of intermittent versus daily vitamin D supplementation on improving circulating 25(OH)D concentration: a Bayesian network meta-analysis of randomized controlled trials
Source: Front Nutr. 2023 Aug 24;10:1168115. doi: 10.3389/fnut.2023.1168115 (PMC10488712; doi:10.3389/fnut.2023.1168115)
Supplement: Supplementary file 10 [file Table_10.DOCX]

| Table S5. Sensitivity analysis of vitamin D supplementation method on 25(OH)D concentration for total 100,000 IU supplementation during two months. | | | | | | | | | | |
| --- | --- | --- | --- | --- | --- | --- | --- | --- | --- | --- |
| Variables |  |  | Daily | |  | Weekly | |  | Monthly | |
|  |  |  | N | Pooled MD, nmol/L |  | N | Pooled MD, nmol/L |  | N | Pooled MD, nmol/L |
| Mean Age | < 60 years |  | 2 | 29(-1,59) |  | 1 | 38(-14,89) |  | 3 | 30(5,53) |
|  | ≥ 60 years |  | 2 | 32(-15,77) |  | 1 | 13(-52,77) |  | 2 | 23(-21,70) |
| Male Proportion | < 50% |  | 4 | 32(13,50) |  | 1 | 18(-21,55) |  | 4 | 31(13,50) |
|  | ≥ 50% |  | NA | |  | NA | |  | 1 | 12(-5,29) |
|  | Unclear |  | NA | |  | 1 | 38(-18,93) |  | NA | |
| Location | Asia |  | 2 | 35(1,67) |  | 1 | 23(-25,69) |  | 2 | 40(7,73) |
|  | Europe |  | NA | |  | 1 | 38(-7,83) |  | 2 | 12(-13,38) |
|  | America |  | 2 | 33(-5,69) |  | NA | |  | 1 | 36(-16,88) |
| Detecting method of 25(OH)D | LC-MS |  | NA | |  | NA | |  | 2 | 12(1,23) |
|  | ELISA |  | NA | |  | NA | |  | 1 | 36(-10,82) |
|  | Chemiluminescence |  | 1 | 35(-14,84) |  | 1 | 38(-20,94) |  | 1 | 39(-11,89) |
|  | Radioimmunoassay |  | 1 | 42(-11,96) |  | NA | |  | NA | |
|  | Others |  | 2 | 22(-8,52) |  | 1 | 11(-34,55) |  | 1 | 29(-17,74) |
| Baseline population disease status | No |  | 2 | 34(-5,69) |  | NA | |  | NA | |
|  | Yes |  | 2 | 32(2,61) |  | 2 | 23(-9,56) |  | 5 | 28(10,47) |
| Baseline vitamin D deficiency | No |  | 4 | 32(14,48) |  | 2 | 21(-7,50) |  | 3 | 18(0,41) |
|  | Yes |  | NA | |  | NA | |  | 2 | 37(12,63) |
| N indicates number of trials; MD indicates mean difference. LC-MS indicates liquid chromatography mass spectrometry. ELISA indicates enzyme-linked immunosorbent assay. | | | | | | | | | | |

| Table S6. Sensitivity analysis of vitamin D supplementation method on 25(OH)D concentration for total 200,000 IU supplementation during two months. | | | | | | | | |
| --- | --- | --- | --- | --- | --- | --- | --- | --- |
| Variables | |  | Daily | |  | Weekly | | |
|  |  |  | N | Pooled MD, nmol/L |  | N | Pooled MD, nmol/L | |
| Mean Age | < 60 years |  | 4 | 32(17,50) |  | 2 | 33(10,56) | |
|  | ≥ 60 years |  | NA | |  | 3 | 58(-8,124) | |
| Male Proportion | < 50% |  | 1 | 41(-63,144) |  | 4 | 51(-1,103) | |
|  | ≥ 50% |  | 3 | 29(6,57) |  | 1 | 39(-3,81) | |
| Location | Asia |  | 2 | 25(3,48) |  | 2 | 33(11,56) | |
|  | Europe |  | 1 | 44(-14,101) |  | 1 | 39(-17,95) | |
|  | America |  | 1 | 41(-82,165) |  | 1 | 97(-26,220) | |
|  | Unclear |  | NA | |  | 1 | 39(-11,88) | |
| Latitude | < 30^o^ |  | NA | | | | | |
|  | 30^o^- 59^o^ |  | 4 | 34(-4,72) |  | 3 | 56(12,98) | |
|  | ≥ 60^o^ |  | NA | |  | 2 | 39(12,65) | |
| Detecting method of 25(OH)D | LC-MS |  | 1 | 44(-80,168) |  | 1 | 98(-25,220) | |
|  | ELISA |  | 1 | 26(-18,70) |  | 2 | 33(5,61) | |
|  | Chemiluminescence |  | 1 | 25(-24,74) |  | 1 | 39(-11,88) | |
|  | Radioimmunoassay |  | NA | | | | | |
|  | Others |  | 1 | 41(-12,94) |  | NA | | |
|  | Unclear |  | NA | |  | 1 | 39(-11,89) | |
| Baseline population disease status | No |  | 1 | 41(-12,94) |  | NA | | |
|  | Yes |  | 2 | 25(-28,79) |  | 5 | 49(15,82) | |
|  | Unclear |  | 1 | 44(-14,101) |  | NA | | |
| Baseline vitamin D deficiency | No |  | 3 | 31(-8,70) |  | 5 | | 49(19,78) |
|  | Yes |  | 1 | 44(-14,101) |  | NA | | |
| N indicates number of trials; MD indicates mean difference. LC-MS indicates liquid chromatography mass spectrometry; ELISA indicates enzyme-linked immunosorbent assay. | | | | | | | | |

| Table S7. Sensitivity analysis of vitamin D supplementation method on 25(OH)D concentration for total 90,000 IU supplementation during three months. | | | | | | | | | |
| --- | --- | --- | --- | --- | --- | --- | --- | --- | --- |
| Variables |  |  | Daily | |  | Weekly | | | |
|  |  |  | N | Pooled MD, nmol/L |  | N | Pooled MD, nmol/L | | |
| Mean Age | < 60 years |  | 16 | 33(21,44) | | 1 | 37(-6,80) | | |
|  | ≥ 60 years |  | NA | |  | 2 | 12(-12,36) | | |
|  | Unclear |  | 2 | 29(-1,58) |  | NA | | | |
| Male Proportion | < 50% |  | 16 | 32(22,43) | | 3 | 21(-2,44) | | |
|  | ≥ 50% |  | 2 | 19(-5,39) | | NA | | | |
| Location | Asia |  | 9 | 31(22,41) | | NA | | | |
|  | Europe |  | 2 | 24(0,48) | | 2 | | | 27(2,51) |
|  | America |  | 5 | 26(5,44) | | NA | | | |
|  | Unclear |  | 2 | 52(-30,131) | | 1 | | 2(-112,116) | |
| Latitude | < 30^o^ |  | 2 | 29(2,58) | | 1 | 2(-37,41) | | |
|  | 30^o^- 59^o^ |  | 14 | 29(22,36) | | 2 | 30(10,49) | | |
|  | ≥ 60^o^ |  | NA | | | | | | |
|  | Unclear |  | 2 | 52(-30,131) |  | NA | | | |
| Co-supplementation with Calcium | Yes |  | 3 | 34(21,48) |  | NA | | | |
|  | No |  | 15 | 30(18,41) | | 3 | 20(-5,46) | | |
| Detecting method of 25(OH)D | LC-MS |  | 4 | 43(-4,88) |  | NA | | | |
|  | ELISA |  | 6 | 30(13,46) |  | NA | | | |
|  | Chemiluminescence |  | 2 | 24(-11,58) | | 3 | 18(-11,46) | | |
|  | Radioimmunoassay |  | 3 | 25(-3,46) |  | NA | | | |
|  | Others |  | 3 | 28(-1,58) |  | NA | | | |
| Baseline population disease status | No |  | 9 | 28(17,37) |  | 1 | 24(-4,52) | | |
|  | Yes |  | 7 | 39(15,63) |  | 1 | 2(-62,65) | | |
|  | Unclear |  | 2 | 25(-8,58) | | 1 | 30(-16,75) | | |
| Baseline vitamin D deficiency | No |  | 16 | 33(22,43) | | 2 | 12(-16,41) | | |
|  | Yes |  | 2 | 25(-8,57) | | 1 | 30(-16,75) | | |
| N indicates number of trials; MD indicates mean difference. LC-MS indicates liquid chromatography mass spectrometry; ELISA indicates enzyme-linked immunosorbent assay. | | | | | | | | | |

| Table S8. Sensitivity analysis of vitamin D supplementation method on 25(OH)D concentration for total 180,000 IU supplementation during three months. | | | | | | | |
| --- | --- | --- | --- | --- | --- | --- | --- |
| Variables |  |  | Daily | |  | Monthly | |
|  |  |  | N | Pooled MD, nmol/L |  | N | Pooled MD, nmol/L |
| Mean Age | < 60 years |  | 16 | 39(29,49) |  | 3 | 38(13,66) |
|  | ≥ 60 years |  | 2 | 24(-4,52) |  | 1 | 16(-22,54) |
| Male Proportion | < 50% |  | 14 | 41(30,51) |  | 3 | 41(14,69) |
|  | ≥ 50% |  | 4 | 21(6,41) |  | 1 | 16(-17,48) |
| Location | Asia |  | 7 | 37(14,59) |  | 2 | 60(14,112) |
|  | Europe |  | 2 | 34(5,62) |  | NA | |
|  | America |  | 5 | 37(11,61) |  | 1 | 16(-39,71) |
|  | Oceania |  | NA | |  | 1 | 16(-11,42) |
|  | Unclear |  | 4 | 44(27,56) |  | NA | |
| Latitude | < 30^o^ |  | NA | |  | 2 | 35(-15,82) |
|  | 30^o^- 59^o^ |  | 14 | 36(25,48) |  | 2 | 30(-6,70) |
|  | ≥ 60^o^ |  | NA | | | | |
|  | Unclear |  | 4 | 44(27,56) |  | NA | |
| Co-supplementation with Calcium | Yes |  | 1 | 54(-14,123) |  | NA | |
|  | No |  | 17 | 37(27,46) |  | 4 | 32(11,54) |
| Detecting method of 25(OH)D | LC-MS |  | 1 | 15(-5,33) |  | NA | |
|  | ELISA |  | 7 | 43(21,63) |  | 1 | 89(1,175) |
|  | Chemiluminescence |  | 2 | 43(-3,90) |  | NA | |
|  | Radioimmunoassay |  | 3 | 43(12,67) |  | NA | |
|  | Others |  | 4 | 32(2,61) |  | 2 | 33(-8,75) |
|  | Unclear |  | 1 | 33(10,55) |  | 1 | 35(-5,73) |
| Baseline population disease status | No |  | 6 | 40(28,51) |  | 2 | 28(-5,66) |
|  | Yes |  | 12 | 36(25,47) |  | 2 | 32(10,56) |
| Baseline vitamin D deficiency | No |  | 15 | 33(9,58) |  | 4 | 34(7,61) |
|  | Yes |  | 3 | 46(31,60) |  | NA | |
| N indicates number of trials; MD indicates mean difference. LC-MS indicates liquid chromatography mass spectrometry; ELISA indicates enzyme-linked immunosorbent assay. | | | | | | | |

| Table S9. Sensitivity analysis of vitamin D supplementation method on 25(OH)D concentration for total 300,000 IU supplementation during three months. | | | | | | | | |
| --- | --- | --- | --- | --- | --- | --- | --- | --- |
| Variables |  |  | Daily | | |  | Weekly | |
|  |  |  | N | Pooled MD, nmol/L | |  | N | Pooled MD, nmol/L |
| Mean age | < 60 years |  | 11 | 34(19,49) | | | 8 | 31(14,48) |
|  | ≥ 60 years |  | NA | | | | 1 | 58(-16,132) |
| Male proportion | < 50% |  | 8 | 39(16,62) | | | 4 | 39(7,72) |
|  | ≥ 50% |  | 3 | 19(2,38) | | | 4 | 32(17,47) |
|  | Unclear |  | NA | | |  | 1 | 21(-6,48) |
| Location | Asia |  | 3 | | 22(3,40) |  | 5 | 28(14,43) |
|  | Europe |  | 2 | | 40(3,75) |  | 2 | 35(-1,70) |
|  | America |  | 4 | | 53(2,103) |  | 1 | 58(-43,160) |
|  | Africa |  | 1 | | 3(-2,7) |  | NA | |
|  | Unclear |  | 1 | | 18(-32,67) |  | 1 | 37(-12,85) |
| Latitude | < 30^o^ |  | 1 | 3(-42,47) | |  | 1 | 35(-10,80) |
|  | 30^o^- 59^o^ |  | 9 | 39(22,56) | | | 7 | 34(14,53) |
|  | ≥ 60^o^ |  | NA | | | | | |
|  | Unclear |  | 1 | 18(-32,68) | |  | 1 | 37(-12,85) |
| Co-supplementation with calcium | Yes |  | 1 | 81(-21,183) | |  | 1 | 58(-43,160) |
|  | No |  | 10 | 29(16,42) | | | 8 | 31(16,46) |
| Detecting method of 25(OH)D | LC-MS |  | 3 | 26(-8,62) | |  | 3 | 43(8,77) |
|  | ELISA |  | 3 | 15(-3,33) | |  | 4 | 26(10,42) |
|  | Chemiluminescence |  | 1 | 81(-21,182) | |  | 1 | 36(-67,138) |
|  | Radioimmunoassay |  | 1 | 18(-12,48) | |  | NA | |
|  | Others |  | 2 | 58(-18,135) | |  | NA | |
|  | Unclear |  | 1 | 34(-13,80) | | | 1 | 37(-10,84) |
| Baseline population disease status | No |  | 4 | 57(17,97) | |  | 2 | 35(-21,92) |
|  | Yes |  | 7 | 20(8,34) | | | 7 | 34(21,47) |
| Baseline vitamin D deficiency | No |  | 9 | 35(17,53) | | | 7 | 33(13,54) |
|  | Yes |  | 2 | 29(-10,71) | | | 2 | 36(-4,76) |
| N indicates number of trials; MD indicates mean difference. LC-MS indicates liquid chromatography mass spectrometry; ELISA indicates enzyme-linked immunosorbent assay. | | | | | | | | |

| Table S10. Sensitivity analysis of vitamin D supplementation method on 25(OH)D concentration for total 600,000 IU supplementation during three months. | | | | | | | |
| --- | --- | --- | --- | --- | --- | --- | --- |
| Variables |  |  | Daily | |  | Weekly | |
|  |  |  | N | Pooled MD, nmol/L |  | N | Pooled MD, nmol/L |
| Mean Age | < 60 years |  | 1 | 50(-20,121) |  | 17 | 60(43,77) |
|  | ≥ 60 years |  | 1 | 100(26,173) |  | 5 | 74(41,107) |
| Male Proportion | < 50% |  | 1 | 50(-17,118) |  | 16 | 70(53,87) |
|  | ≥ 50% |  | 1 | 100(40,160) |  | 5 | 37(13,66) |
|  | Unclear |  | NA | |  | 1 | 66(-18,151) |
| Location | Asia |  | 1 | 51(-21,122) |  | 17 | 63(45,81) |
|  | Europe |  | 1 | 100(-1,200) |  | 2 | 51(-19,122) |
|  | America |  | NA | |  | 2 | 88(23,154) |
|  | Africa |  | NA | |  | 1 | 41(-11,93) |
| Latitude | < 30^o^ |  | 1 | 50(-6,106) |  | 2 | 43(3,89) |
|  | 30^o^- 59^o^ |  | NA | |  | 20 | 65(50,80) |
|  | ≥ 60^o^ |  | 1 | 99(-27,225) |  | NA | |
| Co-supplementation with Calcium | Yes |  | NA | |  | 1 | 41(-11,93) |
|  | No |  | 2 | 74(28,121) |  | 21 | 64(50,79) |
| Detecting method of 25(OH)D | LC-MS |  | 1 | 100(-25,224) |  | NA | |
|  | ELISA |  | NA | |  | 9 | 63(32,94) |
|  | Chemiluminescence |  | 1 | 51(-30,129) |  | 7 | 66(36,96) |
|  | Radioimmunoassay |  | NA | | | | |
|  | Others |  | NA | |  | 1 | 62(-17,142) |
|  | Unclear |  | NA | |  | 5 | 57(17,100) |
| Baseline population disease status | No |  | NA | | | | |
|  | Yes |  | 2 | 74(26,123) |  | 19 | 63(47,79) |
|  | Unclear |  | NA | |  | 3 | 65(8,120) |
| Baseline vitamin D deficiency | No |  | 2 | 74(11,139) |  | 9 | 71(40,101) |
|  | Yes |  | NA | |  | 13 | 58(42,74) |
| N indicates number of trials; MD indicates mean difference. LC-MS indicates liquid chromatography mass spectrometry; ELISA indicates enzyme-linked immunosorbent assay. | | | | | | | |

| Table S11. Sensitivity analysis of vitamin D supplementation method on 25(OH)D concentration for total 300,000 IU supplementation during six months. | | | | | | | |
| --- | --- | --- | --- | --- | --- | --- | --- |
| Variables |  |  | Daily | |  | Monthly | |
|  |  |  | N | Pooled MD, nmol/L |  | N | Pooled MD, nmol/L |
| Mean Age | < 60 years |  | 2 | 31(-6,70) |  | 1 | 25(-30,79) |
|  | ≥ 60 years |  | 4 | 34(22,48) |  | 2 | 35(13,49) |
|  | Unclear |  | NA | |  | 1 | 15(-5,36) |
| Male Proportion | < 50% |  | 1 | 36(-4,75) |  | 2 | 22(-8,50) |
|  | ≥ 50% |  | 5 | 33(17,50) |  | 2 | 27(4,51) |
| Location | Asia |  | 1 | 37(-14,87) |  | 1 | 15(-32,63) |
|  | Europe |  | 4 | 33(18,49) |  | 1 | 40(10,70) |
|  | America |  | 1 | 33(-13,79) |  | NA | |
|  | Oceania |  | NA | |  | 2 | 22(-1,44) |
| Latitude | < 30^o^ |  | NA | |  | 1 | 18(-12,46) |
|  | 30^o^- 59^o^ |  | 4 | 36(21,53) |  | 3 | 27(10,44) |
|  | ≥ 60^o^ |  | 2 | 28(-1,58) |  | NA | |
| Co-supplementation with Calcium | Yes |  | 1 | 36(-11,82) |  | NA | |
|  | No |  | 5 | 33(20,46) |  | 4 | 25(11,39) |
| Detecting method of 25(OH)D | LC-MS |  | 2 | 28(-2,58) |  | NA | |
|  | ELISA |  | 1 | 37(-14,87) |  | NA | |
|  | Chemiluminescence |  | NA | |  | 2 | 19(-1,41) |
|  | Radioimmunoassay |  | 1 | 34(-9,77) |  | NA | |
|  | Others |  | 1 | 33(-20,86) |  | 1 | 40(-11,91) |
|  | Unclear |  | 1 | 44(-13,101) |  | 1 | 17(-41,76) |
| Baseline population disease status | No |  | 3 | 34(22,46) |  | 2 | 22(5,39) |
|  | Yes |  | 3 | 32(6,61) |  | 1 | 40(-5,85) |
|  | Unclear |  | NA | |  | 1 | 15(-5,36) |
| Baseline vitamin D deficiency | No |  | 5 | 35(27,46) |  | 3 | 32(18,42) |
|  | Yes |  | 1 | 21(-8,49) |  | 1 | 15(-11,42) |
| N indicates number of trials; MD indicates mean difference. LC-MS indicates liquid chromatography mass spectrometry; ELISA indicates enzyme-linked immunosorbent assay. | | | | | | | |

| Table S12. Sensitivity analysis of vitamin D supplementation method on 25(OH)D concentration for total 600,000 IU supplementation during six months. | | | | | | | | |
| --- | --- | --- | --- | --- | --- | --- | --- | --- |
| Variables |  | |  | Daily | |  | Weekly | |
|  |  |  |  | N | Pooled MD, nmol/L |  | N | Pooled MD, nmol/L |
| Mean Age | < 60 years | |  | 1 | 37(-6,80) | | 8 | 41(26,56) |
|  | ≥ 60 years | |  | 1 | 69(23,115) |  | 5 | 47(27,68) |
| Male Proportion | < 50% | |  | 1 | 37(-14,88) | | 8 | 43(25,61) |
|  | ≥ 50% | |  | 1 | 69(48,90) |  | 5 | 45(35,54) |
| Location | Asia | |  | NA | |  | 8 | 45(30,59) |
|  | Europe | |  | 1 | 69(21,117) |  | 3 | 37(7,63) |
|  | America | |  | NA | |  | 2 | 51(-10,114) |
|  | Oceania | |  | 1 | 37(-11,86) |  | NA | |
| Latitude | < 30^o^ | |  | NA | |  | 1 | 51(-14,115) |
|  | 30^o^- 59^o^ | |  | 2 | 54(26,82) | | 11 | 43(31,55) |
|  | ≥ 60^o^ | |  | NA | |  | 1 | 42(-11,96) |
| Co-supplementation with Calcium | Yes | |  | NA | |  | 1 | 70(-19,158) |
|  | No | |  | 2 | 54(31,78) | | 12 | 41(32,51) |
| Detecting method of 25(OH)D | LC-MS | |  | NA | |  | 4 | 41(8,75) |
|  | ELISA | |  | NA | |  | 4 | 36(23,50) |
|  | Chemiluminescence | |  | NA | |  | 2 | 46(11,82) |
|  | Radioimmunoassay | |  | 1 | 69(-18,155) |  | NA | |
|  | Others | |  | 1 | 38(-49,124) | | 3 | 53(4,103) |
| Baseline population disease status | No | |  | 1 | 69(41,97) | | 5 | 40(26,52) |
|  | Yes | |  | 1 | 37(-14,89) | | 7 | 48(29,68) |
|  | Unclear | |  | NA | |  | 1 | 32(-9,72) |
| Baseline vitamin D deficiency | No |  | | 1 | 69(29,109) | | 10 | 44(31,57) |
|  | Yes | |  | 1 | 37(-11,86) | | 3 | 42(14,70) |
| N indicates number of trials; MD indicates mean difference. LC-MS indicates liquid chromatography mass spectrometry; ELISA indicates enzyme-linked immunosorbent assay. | | | | | | | | |

| Table S13. Sensitivity analysis of vitamin D supplementation method on 25(OH)D concentration for total 720,000 IU supplementation during twelve months. | | | | | | | |
| --- | --- | --- | --- | --- | --- | --- | --- |
| Variables |  |  | Daily | |  | Monthly | |
|  |  |  | N | Pooled MD, nmol/L |  | N | Pooled MD, nmol/L |
| Mean Age | < 60 years |  | 3 | 46(23,73) |  | 1 | 23(-17,62) |
|  | ≥ 60 years |  | 3 | 39(23,53) |  | 2 | 31(11,47) |
|  |  |  | NA | |  | 1 | 11(-4,26) |
| Male Proportion | < 50% |  | 4 | 40(29,52) |  | 1 | 23(0,45) |
|  | ≥ 50% |  | 1 | 41(-2,84) |  | 2 | 16(-15,47) |
|  | Unclear |  | 1 | 55(-23,133) |  | 1 | 36(-34,106) |
| Location | Asia |  | NA | |  | 1 | 11(-5,26) |
|  | Europe |  | 3 | 41(26,57) |  | 2 | 22(5,39) |
|  | America |  | 2 | 41(9,74) |  | NA | |
|  | Oceania |  | NA | |  | 1 | 36(-10,82) |
|  | Africa |  | 1 | 44(-13,101) |  | NA | |
| Latitude | < 30^o^ |  | 1 | 44(-14,101) |  | 1 | 36(-19,92) |
|  | 30^o^- 59^o^ |  | 4 | 41(33,51) |  | 3 | 18(8,28) |
|  | ≥ 60^o^ |  | 1 | 35(-11,81) |  | NA | |
| Co-supplementation with Calcium | Yes |  | 1 | 35(-10,81) |  | NA | |
|  | No |  | 5 | 42(32,54) |  | 4 | 23(12,35) |
| Detecting method of 25(OH)D | LC-MS |  | 2 | 41(18,63) |  | 2 | 22(-1,45) |
|  | ELISA |  | NA | | | | |
|  | Chemiluminescence |  | 1 | 37(-13,86) |  | 2 | 24(-11,59) |
|  | Radioimmunoassay |  | 3 | 42(20,71) |  | NA | |
|  | Others |  | NA | | | | |
| Baseline population disease status | No |  | 3 | 41(24,57) |  | 1 | 36(8,65) |
|  | Yes |  | 3 | 40(26,60) |  | 2 | 22(4,41) |
|  | Unclear |  | NA | |  | 1 | 11(-4,26) |
| Baseline vitamin D deficiency | No |  | 4 | 42(31,52) |  | 3 | 29(16,39) |
|  | Yes |  | 2 | 41(-1,91) |  | 1 | 11(-50,72) |
| N indicates number of trials; MD indicates mean difference. LC-MS indicates liquid chromatography mass spectrometry; ELISA indicates enzyme-linked immunosorbent assay. | | | | | | | |
